# *Clostridium butyricum* and its metabolites regulate macrophage polarization through miR-146a to antagonize gouty arthritis

**DOI:** 10.1016/j.jare.2025.05.036

**Published:** 2025-05-19

**Authors:** Siyue Song, Kaiyue Shi, Moqi Fan, Xianghui Wen, Jiatao Li, Yining Guo, Yu Lou, Fusen Chen, Jialu Wang, Lin Huang, Chengping Wen, Tiejuan Shao

**Affiliations:** a College of Basic Medical Sciences, Zhejiang Chinese Medical University, Hangzhou 310053, China; b Center for Innovative Basic Research in Autoimmune Diseases in Medicine, Hangzhou 310053, China; c The Second Clinical Medical College, Zhejiang Chinese Medical University, Hangzhou 310053, China

**Keywords:** Gouty arthritis, *Clostridium butyricum*, Macrophage polarization, miR-146a, SOCS7/JAK2-STAT3 pathway

## Abstract

**Introduction:**

Gut microbiota modulation has recently been identified as a prospective avenue for the exploration of novel therapeutic strategies for the management of gout. Nevertheless, the application of a single specific strain or bacterial metabolite for gout intervention has rarely been explored and the underlying regulatory mechanism remains elusive.

**Objectives:**

To ascertain the potential role and the molecular mechanism of *Clostridium butyricum* and butyrate in the management of gouty arthritis.

**Methods:**

A *Uox*-KO mouse model of gouty arthritis was developed and the composition of the gut microbiota was analyzed. *C. butyricum* and butyrate were supplemented to assess functional recovery and intestinal homeostasis. NanoString analysis identified miRNA variations. GC/MS measured butyric acid levels and qPCR detected the abundance of butyrate-producing enzymes and bacteria. Flow cytometry analyzed macrophage polarization and ELISA measured pro-inflammatory cytokine production. Agomir and antagomir were transfected and dual-luciferase reporter assay was adapted for validation of miRNA target binding. siRNA and rescue experiments were performed to validate the role of SOCS7 in macrophage polarization. In addition, a cohort of patients with gouty arthritis were assembled for the purpose of validating the molecular mechanism.

**Results:**

The results of our study demonstrated that a reduction of butyrate levels, resulting from a deficiency of butyrate-producing bacteria, leads to aberrant miR-146a expression. This, in turn, induces an imbalance in macrophage polarization and the onset of gouty arthritis. The administration of *C. butyricum* and butyrate demonstrated considerable anti-inflammatory efficacy by restoring intestinal homeostasis, modulating miR-146a expression, and skewing macrophage polarization. The SOCS7/JAK2-STAT3 signaling pathway was identified as a pivotal mediator in the skewing of macrophage polarization induced by miR-146a.

**Conclusion:**

Our findings enrich the understanding of the regulatory mechanisms underlying macrophage polarization in gouty arthritis and highlight the potential applications of probiotics and their metabolites in clinical gout treatment.

## Highlights

• Reduction in butyrate-producing bacteria contributes to gouty arthritis.
• Supplementation of C. *butyricum* and butyrate provides a promising therapeutic avenue for the treatment of gouty arthritis.
• C. *butyricum* and butyrate regulate macrophage polarization to antagonize gouty arthritis.
• *C. butyricum* and butyrate modulate macrophage polarization by regulating miR-146a expression.
• miR-146a orchestrates macrophage polarization through the SOCS7/JAK2-STAT3 signaling pathway.

## Graphical abstract

**Figure:**
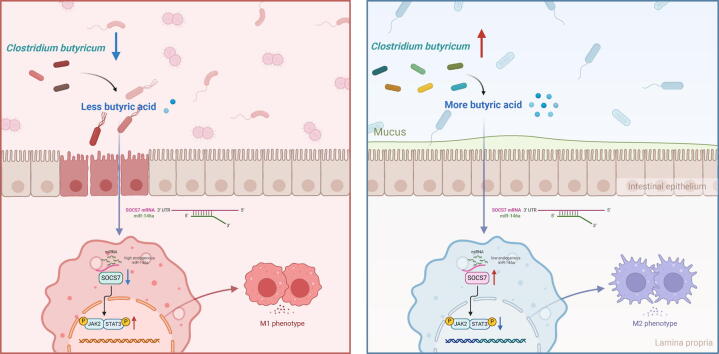

## Introduction

Gout is a prevalent chronic inflammatory arthropathy, defined by hyperuricemia and the deposition of monosodium urate (MSU) crystals in articular and periarticular tissues. It affects an estimated 0.68 % to 3.90 % of the adult population globally and results in a high comorbidity burden [1,2]. Gout entails a multitude of intricate complications, including impaired joint function, hypertension, obesity, type 2 diabetes, cardiovascular events, and chronic kidney disease [3,4]. It is currently acknowledged that macrophage exerts a pivotal influence on the onset and resolution of gouty arthropathy, and the infiltration of M1 macrophages has been identified as a positive correlate of arthritis severity. Regulation of macrophage polarization has therefore been implicated as a key element in the pathology of gouty arthritis [5,6].

Despite the increasing understanding of the factors that regulate the inflammatory response, the influence of the gut microbiome on gout remains to be fully elucidated. Previous research has indicated the correlation between gut microbiota imbalance and dysregulated host urate catabolism and systemic inflammation [7]. Targeting the composition and metabolism of intestinal flora has attracted considerable interest for gout therapy. However, the underlying mechanism by which the gut flora affects gout remains unclear. Herein, the current work examined the impact of *Clostridium butyricum* and its metabolite butyrate on the progression of gouty inflammation, with a particular emphasis on macrophage polarization.

MicroRNAs (miRNAs) are a type of small non-coding endogenous RNA molecule that regulates gene expression by repressing translation or degrading mRNA. There is evidence that miRNAs are involved in a variety of inflammatory diseases, including gout, systemic lupus erythematosus, rheumatoid arthritis, inflammatory bowel disease, and psoriasis [[7], [8], [9]]. In recent years, miRNAs have emerged as pivotal regulators in modulating not only macrophage activation [10] and polarization [11], but also their inflammation resolution [12]. miR-146a, a miRNA found to be involved in negative feedback regulation of inflammation and immune response, has been demonstrated to be associated with disease severity in gout patients [12,13]. Intervention with miR-146a is a potential mechanism to alleviate inflammation in gout by modulating inflammation and immune cells. In addition, miRNAs have been identified as crucial molecular mediators in the mediation of intestinal flora-host interactions, and intestinal flora and its metabolites have been found to regulate host miRNA gene expression [14]. However, the mechanism of miR-146a in the progression of gout inflammatory processes and the role of intestinal flora in the modulation of miR-146a need to be further investigated.

Given that macrophages exhibit functional plasticity under the stimulation of various microenvironmental factors, we investigated the communication between gut microbiota and macrophages through miR-146a. In this study, we evaluated the potential therapeutic effects of *C. butyricum* and butyrate on gouty arthritis by employing *Uox*-KO mice, an optimal model that is extensively utilized for investigations pertaining to hyperuricemia and gout [[15], [16], [17]]. We confirmed the regulatory mechanism of butyrate on macrophage polarization by modulating miR-146a expression in *vivo* and in *vitro*. We also made some observations and validations on clinical patients. Our study aimed to elucidate the underlying mechanism involving macrophage polarization and identify potential therapeutic targets for the prophylaxis and treatment of gouty arthritis.

## Materials and methods

### Animals

*Uox*-KO mice were supplied by Shanghai Southern Model Biotechnology Co., Ltd and all mice used in the experiment were housed in standard environmental conditions. The *C. butyricum* intervention group was gavaged with 10^9^ CFU/mL strain for 6 weeks, and the sodium butyrate intervention group was gavaged with 500 mg/kg/day sodium butyrate for 6 weeks. The *Uox*-KO mice were injected with 25 mg/mL MSU crystals into the right footpad 24 h before sacrifice. To evaluate the condition of the mice, serum uric acid (SUA) and urinary uric acid (UUA) levels were examined using an automated biochemical analyzer, and the swelling index and mechanical pain threshold were assessed as previously reported [18,19].

### Donor cohorts

Prior to their participation, an informed consent form was signed by all participants. The study population exclusively comprised males aged between 25 and 47 years. Confirmed healthy (n = 45) and gouty arthritis (n = 62) subjects were enrolled for the study of butyric acid concentration, the expression of *Buk* and *But,* and the detection of *C. butyricum* abundance. Five subjects from each group were selected for the isolation of peripheral blood mononuclear cells (PBMCs).

### Ethics statement

All the animal experiments were conducted in accordance with the ethical policies and procedures approved by the Laboratory Animal Management and Welfare Ethical Review Committee of Zhejiang Chinese Medical University (Approval no. IACUC-20210621-16). All experiments involving human subjects were executed after being approved by the Ethics Committee of Zhejiang Chinese Medical University (Approval no. 20240329-6).

### Cell culture

Macrophages derived from bone marrow (BMDMs) were harvested from the femoral and tibial bones of C57BL/6J mice, ensuring a unique source of these cells for the study. PBMCs were extracted by Ficoll density gradient centrifugation (Cytiva, # 1714403). The cells were cultured in RPMI-1640 medium (supplemented with 10 % FBS, 1 % penicillin–streptomycin solution, and 25 ng/mL M−CSF) for 7 days in a 5 % CO_2_ incubator. Mural adherent cells were collected as BMDMs or PBMC-derived macrophages. The M0 macrophages were stimulated with LPS (100 ng/mL) and IFN-γ (50 ng/mL) or stimulated with MSU (250 µg/mL) for 6 h to induce M1-polarized macrophages. 1.25 mM butyrate was used to evaluate the effect of butyrate in *vitro*. The intestine was cut lengthwise and digested with collagenase I at 37 °C for 1 h on a shaker, then intestinal macrophages were collected by Percoll gradient.

### HE, IHC and IF analysis

Formalin-fixed tissues were dehydrated via ethanol, transparented with xylene, and subsequently embedded in paraffin. 5 µm sections were prepared and stained with H&E for conventional morphological evaluation. The colonic tissues were incubated with primary antibodies against ZO-1 and occludin antibodies to assess the integrity of the gut barrier. Quantitative analysis of the average density of inflammatory infiltrating cells, occludin and ZO-1 expression was performed using ImageJ to assess the tight junction protein levels in the colon tissues of *Uox*-KO mice. The claw section was subjected to immunofluorescence staining with anti-F4/80, anti-iNOS, and anti-CD206 in order to evaluate the ratio of M1 and M2 macrophages. The coverslips were sealed with an anti-fluorescence quenching sealer containing DAPI (Table S1). A quantitative assessment of fluorescence intensity was performed using Image J.

### Flow cytometric analysis

The intestinal and spleen single-cell suspensions were obtained as previously described [18,20]. The cell suspensions were incubated at 4 °C for 30 min with fluorochrome-conjugated antibodies specific for mouse CD45, mouse F4/80, mouse CD86, mouse CD163, or human CD45, human CD68, human CD86, and human CD206 (Table S2) to evaluate the M1/M2 macrophage balance. The data were gathered on a CytoFLEX S flow cytometer (Beckman, USA) and subsequently processed with the FlowJo software (10.4.0).

### 16S rRNA sequencing

Genomic DNA was extracted from colonic samples utilizing the QIAmp DNA Microbiome Kit (Qiagen, Germany), with strict adherence to the manufacturer's guidelines. Subsequently, the V3-V4 region of 16S rRNA gene was amplified and subjected to sequencing on the Illumina NovaSeq PE250 platform. For a more detailed account of the bioinformatic methods employed, please refer to previous studies [18,20]. The raw sequences, which were assigned the accession numbers ranging from SAMN41086027 to SAMN41086040, were deposited in the Sequence Read Archive database.

### Butyrate detection

Butyrate was extracted from acidified fecal suspension and serum using anhydrous ether. For the determination, a combination of gel filtration chromatography-mass spectrometry (GPC-GC/MS-2010, Shimadzu, Japan), equipped with a Rtx-Wax capillary column (Shimadzu, Japan), was employed to provide accurate and reliable results. For a comprehensive account of the test parameters, please refer to the previous reference [16].

### miRNA expression assay

The total RNA was isolated from mice colon with TRIzol reagent (Thermo Fisher Scientific, USA). The colonic miRNA profile was then analyzed using the nanoString quantitative assay platform with nCounter Mouse v1.5 miRNA assay CSO (CSO-MMIR15-12) following the instructions of the manufacturer. The raw miRNA data were subjected to analysis using nSolver Analysis Software v.3.0 (NanoString Technologies, Inc.). The normalization of the data was accomplished through the utilization of positive and negative controls, in conjunction with housekeeping gene probes. The *P* values were adjusted using the false discovery rate (FDR) procedure to identify those miRNAs that were differentially expressed. The miRNAs that satisfied both the criteria of an FDR value below 0.05 and a fold change of greater than 2.0 were deemed to exhibit significantly differential expression.

### miRNA-146a target prediction

The miRNA-146a prediction targets were indexed using the Target Scan database, DIANA database, miRWalk database, and miRDB databases. Gout-related targets were indexed using the ClinVar database, CTD database, DisGeNET database, and GeneCards database. The targets of gout and miRNA-146a were overlapped and the overlapping targets were subsequently analyzed using the STRING database. The PPI network retrieved from the STRING database was visualized by Cytoscape 3.7.2. GO and KEGG pathway enrichment analyses were carried out using the R statistical computing platform (version 4.0.0). The R package “clusterProfiler” was adopted for statistical analysis, with a *P* value less than 0.05 indicating statistical significance.

### Cell viability assay

The cell viability of BMDMs following incubation with varying concentrations of butyrate was determined using the Cell Counting Kit-8 assay (Beyotime, China). The absorbance was then quantified at 450 nm with the use of a plate reader (Thermo Fisher Scientific, USA).

### miRNA transfection

BMDMs were seeded and cultured in 6-well plates. At a confluency of 80 % to 90 %, BMDMs were transfected with 0.5 μg/mL miR-146 agomir, miR-146a antagomir, or an equivalent amount of negative control (NC) mimics using PolyFast Transfection Reagent (MCE, USA). The 2′-O-methyl-modified miR-146a agomir, miR-146a antagomir, NC agomir, and NC antagomir were procured from GenePharma (Shanghai, China). The sequences of these mimics were provided in Table S3 for reference.

### Dual-luciferase reporter assay

The luciferase reporter assay was adapted to validate the target binding of miR-146a and *Socs7*. 293T cells were co-transfected with 100 ng of firefly luciferase reporter vector carrying the wild-type or mutated sequence of the *Socs7* 3′-UTR, 100 ng of miR-146a-3p mimics, 100 ng inhibitor, and 5 ng renilla. Firefly and renilla luciferase levels were quantified 48 h following cotransfection through the utilization of Dual-Glo reporter assay (Promega, USA). The relative firefly luciferase signal was normalized to the corresponding renilla signal.

### *Socs7* knockdown and rescue experiments

To investigate the role of *Socs7* in macrophage polarization and gout pathogenesis, *Socs7* was knocked down in BMDMs using siRNA transfection. Briefly, BMDMs were transfected with 0.5 µg/mL *Socs*7-specific siRNA (*Socs7* siRNA; GenePharma) using PolyFast Transfection Reagent (MCE, USA), and negative control siRNA (NC siRNA) was used as a reference. After 24 h, *Socs7* knockdown efficiency was verified by qPCR. For the rescue experiment, *Socs7* was overexpressed in BMDMs by transfecting 1 µg/mL pcDNA3.1(+) − *Socs7* (designated *Socs7* OE hereafter) or empty pcDNA3.1(+) vector (Vector). *Socs7* overexpression was confirmed by qPCR. All siRNA and plasmid sequences were provided in Table S3.

### Quantitative reverse transcription PCR

Total RNA was extracted from the mouse colon tissue or cultured cells using Trizol reagent, and cDNA was synthesized using the HiFiScript cDNA Synthesis Kit (Cwbio, China). Fecal RNA was extracted from the fecal samples and subsequently converted to cDNA using SuperScript^TM^ IV VILO^TM^ Master Mix (Thermo Fisher Scientific, USA). qPCR was conducted on the LightCycler (Roche, USA) using the SYBR® Green Premix Pro Taq HS qPCR Kit (Accurate Biology, China). Relative miRNA-146a expression was determined using a universal reverse primer with U6 as an internal control. Relative expression of bacterial butyric acid metabolism enzymes or relative abundance of *C. butyricum* was determined with 16S rRNA as a housekeeping gene. Experiments were conducted at least in triplicate. The relative gene expression levels of the target genes were calculated employing the 2^−ΔΔCt^ method. The primers used were listed in Table S4.

### Western blot analysis

Total colon tissue and cellular protein were lysed in RIPA lysis buffer supplemented with protease and phosphatase inhibitors. The protein concentration was subsequently determined, and the targeted proteins were then separated by SDS-PAGE. The separated proteins were then electrotransferred to PVDF membranes and blocked with 5 % BSA for 1  h at room temperature. The blocked membranes were probed with primary antibodies (Table S5) at 4 °C overnight, followed by HRP-conjugated secondary antibodies for 2  h at room temperature. The protein bands were visualized, and the ratios of the target proteins to β-actin were quantified using ImageJ software.

### Quantification of cytokines

Serum, colon tissue homogenates, and the supernatant of BMDMs were collected to measure the concentration of IL-1β, IL-6, and TNF-α. This was done using ELISA kits (Invitrogen, USA) in accordance with the methods described by the manufacturer.

### Bacterial strain and culture

*C. butyricum* was isolated and preserved in our lab, and its identity was confirmed based on colony morphology and sequencing of its 16S rRNA gene, utilizing the universal primers 27F and 1492R. The strain was incubated in MRS at 37 °C, 60 % RH for 24 h in an anaerobic workstation (80 % N_2,_ 10 % H_2_ and 10 % CO_2_). The culture was washed 3 times with sterile PBS before being made into 10^9^ CFU/mL suspension.

### Statistical analysis

The experimental data are presented as mean ± SEM. Statistical comparisons between the two groups were performed utilizing the parametric Student's *t*-test depending on the distribution of the data. For analyses involving multiple groups, the Kruskal-Wallis test or one-way analysis of variance (ANOVA) with Tukey's multiple comparisons test was used. All analyses of the statistical data were conducted with GraphPad Prism (GraphPad, San Diego, CA, USA). Linear discriminant analysis Effect Size (LEfSe) method was used to determine differentially abundant microbes. Spearman’s rank correlation analysis was performed for correlations between the discrepant bacterial genera and gout indictors or the altered miRNA. *P* ＜0.05 was regarded as statistically significant.

## Results

### Imbalanced macrophage polarization in *Uox*-KO mice

**Fig. 1:**
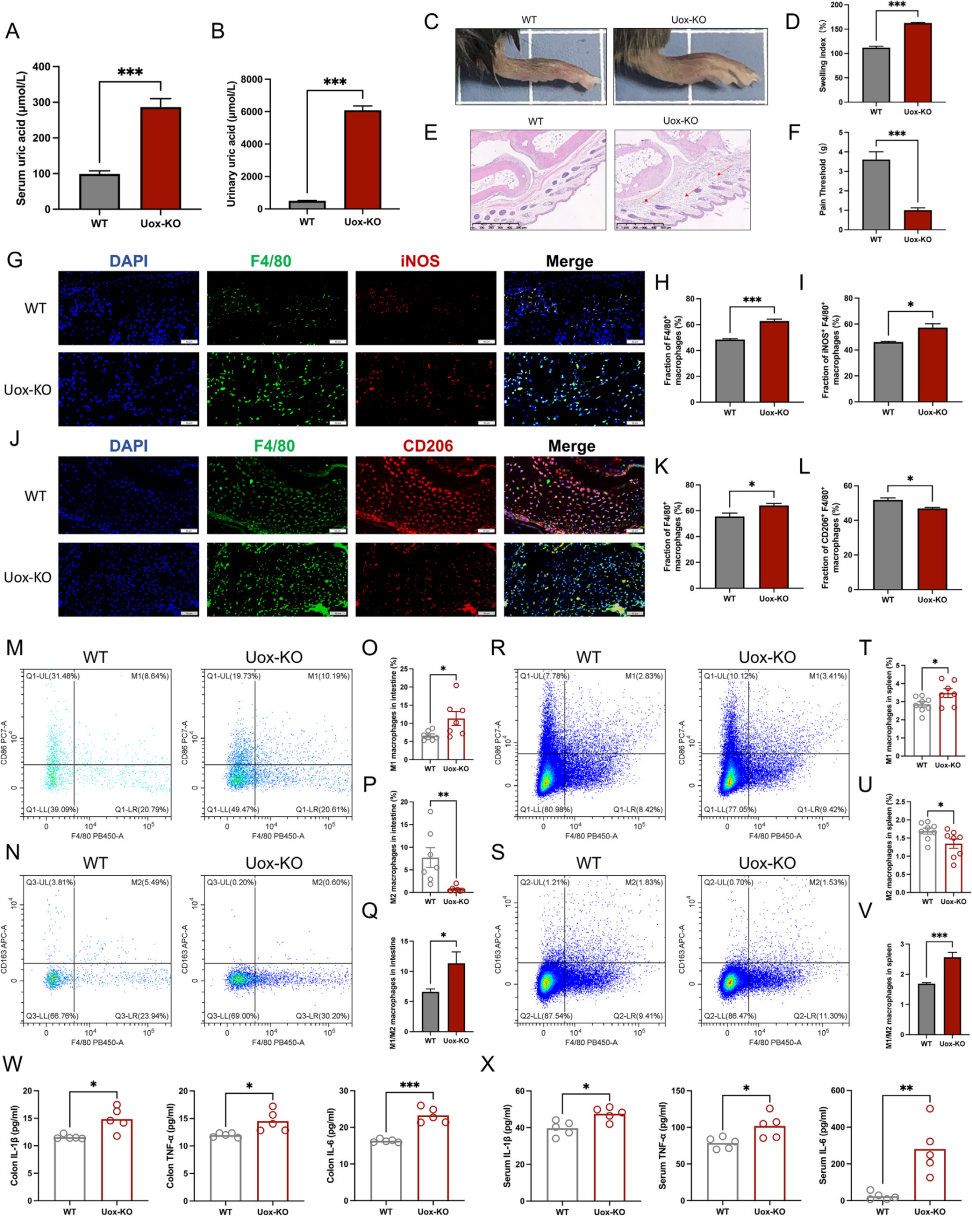
**Altered macrophage polarization in *Uox*-KO mice.** (A) The level of serum uric acid. (B) The level of urinary uric acid. (C-D) Footpad swelling was measured 24 h after MSU crystals injection. (E) Footpad H&E staining. (Scale bar: 500 µm). (F) Mechanical pain threshold. (G-L) Immunofluorescence staining of F4/80, iNOS, and CD206 of footpad. (Scale bar: 50 µm). (M) Representative flow cytometry plots of the intestinal M1 macrophage populations, which were measured as CD86^+^ F4/80^+^ CD45^+^ live cell percentages. (N) Representative flow cytometry plots of the intestinal M2 macrophage populations, which were measured as CD163^+^ F4/80^+^ CD45^+^ live cell percentages. (O-Q) Percentage of intestinal M1 macrophage cells, M2 macrophage cells, and M1/M2 ratio in WT and *Uox*-KO mice. (R-V) Representative flow cytometry plots of the splenic resident M1 and M2 macrophage populations, and percentage of splenic M1 macrophage cells, M2 macrophage cells, and M1/M2 ratio. (W) The concentrations of IL-1β, TNF-α, and IL-6 in colon. (X) The concentrations of serum IL-1β, TNF-α, and IL-6. n = 7–8 mice per group. Values are expressed as mean ± SEM. “ns” represents not significant; *P < 0.05, **P < 0.01, ***P < 0.001.

Consistent with our previous reports, significantly elevated SUA and UUA levels (Fig. 1A–B), damaged renal function (Fig. S1), increased paw pad swelling (Fig. 1C–D) and inflammatory cell infiltration (Fig. 1E), and decreased mechanical pain threshold (Fig. 1F) were observed in *Uox*-KO mice, suggesting the suitability of *Uox*-KO mice as a model to study the pathogenesis of gouty arthritis.

Since macrophages are considered to be the most pivotal effector cells in the development of gouty arthritis [[21], [22], [23]], we examined the macrophage phenotype in the paw pads by immunofluorescence staining on the macrophage marker F4/80, M1 phenotype marker iNOS, and M2 phenotype marker CD206. The results showed a significant increase in the mean fluorescence intensity of M1 (F4/80^+^ iNOS^+^) macrophages (Fig. 1G–I) and a decrease in M2 (F4/80^+^ CD206^+^) macrophages in the paw pads of *Uox*-KO mice compared to WT mice (Fig. 1J–L). Given the function of the gut-joint axis in inflammatory arthritis, we also investigated macrophage phenotypes in the intestine and spleen using flow cytometry. The same changes, increased M1 subtypes and decreased M2 subtypes, were observed in both intestine (Fig. 1M–Q) and spleen (Fig. 1R–V) of *Uox*-KO mice. What's more, the augmented production of IL-1β, IL-6, and TNF-α, in both the intestine (Fig. 1W) and serum (Fig. 1X) of *Uox*-KO mice further highlighted an imbalance in the intestinal macrophage ratio that could trigger intestinal and systemic inflammation.

### Reduction in butyric acid-producing bacteria contributed to gouty arthritis

**Fig. 2:**
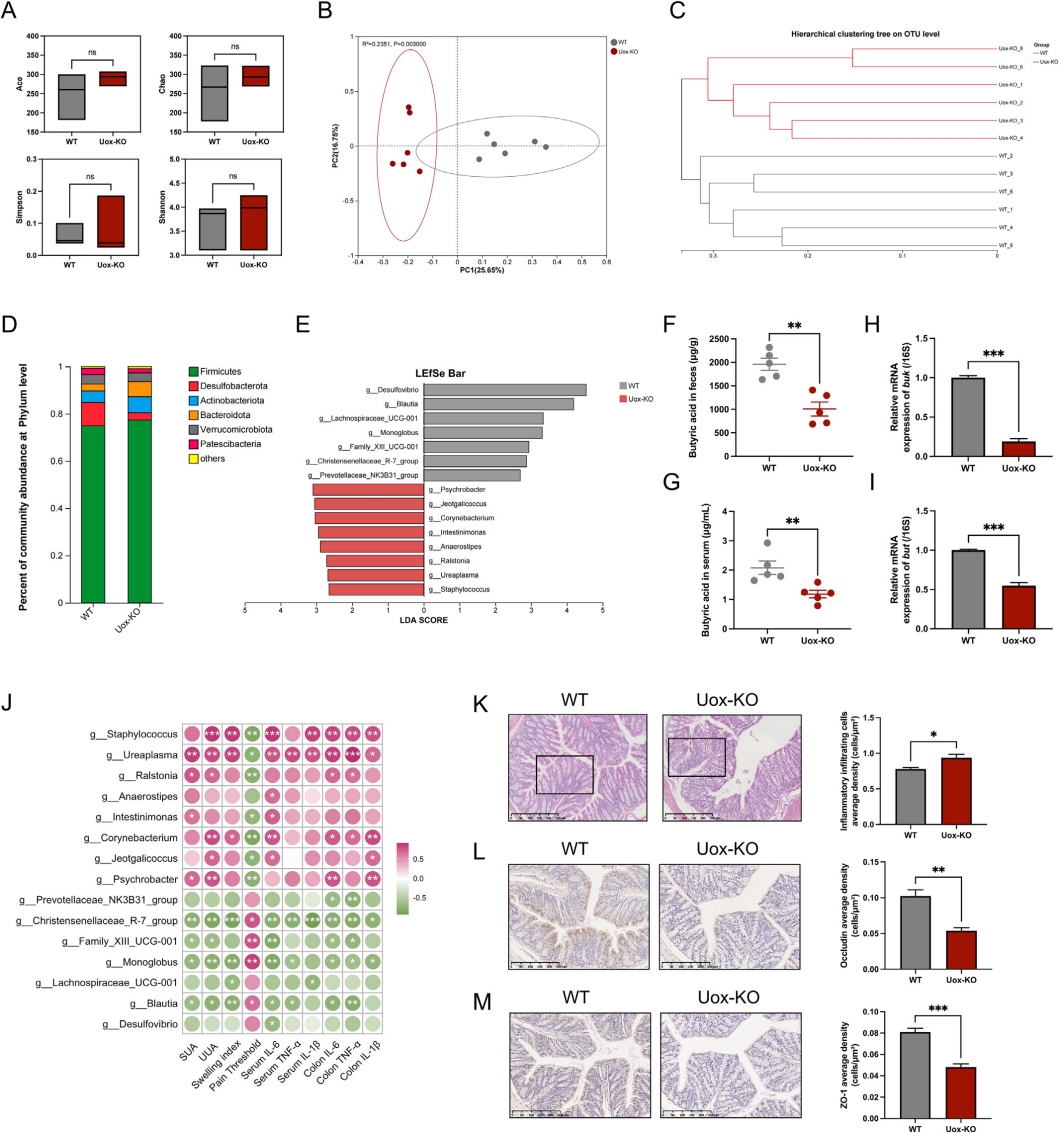
**Butyric acid-producing bacteria decreased in *Uox*-KO mice.** (A) Alpha diversity indicated by ACE, Chao, Shannon, and Simpson. (B) Beta diversity based on principal coordinates analysis (PCoA) (*P* = 0.003). (C) Hierarchical clustering tree of the microbiota by using the average method and Bray-Curtis distances of the two groups. (D) Mean relative abundance of taxa at phylum level. Only the top 6 taxa are listed in the legend. (E) The abundance of differential genera between WT and *Uox*-KO mice calculated by LEfSe analysis. (F-G) Faecal and serum butyric acid concentrations in WT and *Uox*-KO mice. (H-I) Relative expression of *Buk* and *But* in intestinal contents. (J) Spearman’s rank correlation analysis was conducted between the discrepant bacterial genera and gout symptoms. Red shows a positive correlation and green shows a negative correlation. The intensity of the color is directly proportional to the correlation coefficient. *P < 0.05; **P < 0.01; ***P < 0.001. (K) H&E-stained colon sections (Scale bar: 250 µm). (L-M) IHC analysis of occludin and ZO-1 expression in the colon (Scale bar: 250 µm).

Dysbiosis and altered intestinal permeability, the key factors in the pathogenesis of gout, have been shown to induce chronic activation of innate immune cells. Therefore, we further analysed the microbial community composition by amplicon sequencing of 16S rRNA V3-V4 region. The results indicated that, despite the absence of notable discrepancies in alpha diversity between *Uox*-KO and WT mice (Fig. 2A), OTU-level PCoA analysis and hierarchical clustering analysis showed a complete separation, suggesting the differences in the dominant species of gut flora between the two subject groups (Fig. 2B–C). The relative abundance of the Firmicutes and Bacteroidota phylum was increased, while that of Desulfobacterota was decreased in *Uox*-KO mice (Fig. 2D). LEfSe analysis at the genus level showed that the genera *Desulfovibrio*, *Blautia*, *Lachnospiraceae*_UCG-001, *Monoglobus*, Family XIII UCG-001, *Christensenellaceae* R-7 group, and *Prevotellaceae* NK3B31 group were dramatically depleted in *Uox*-KO mice, while *Psychrobacter, Jeotgalicoccus*, *Corynebacterium*, *Intestinimonas*, *Anaerostipes*, *Ralstonia*, *Ureaplasma,* and *Staphylococcus* were significantly enriched (Fig. 2E).

We noted that most of the differential bacteria with significant down-regulation in *Uox*-KO mice, including *Blautia*, *Lachnospiraceae*_UCG-001, *Monoglobus*, *Christensenellaceae* R-7 group, and *Prevotellaceae* NK3B31 group, are butyric acid-producing bacteria. The reduction in butyric acid concentration served to corroborate our findings. Both the intestinal and serum concentrations of butyric acid were significantly reduced in *Uox*-KO mice (Fig. 2F–G). What’s more, the diminished expression of butyrate kinase (*Buk*) and butyryl-CoA: acetyl-CoA transferase (*But*), enzymes that are instrumental in bacterial butyric acid metabolism, substantiated the reduced prevalence of butyric acid-producing bacteria in *Uox*-KO mice (Fig. 2H–I). We further performed Spearman's correlation analysis of these differential strains with gout symptom indicators. The results showed that the downregulated butyric acid-producing bacteria in *Uox*-KO mice exhibited a significant negative correlation with SUA and UUA, swelling index, and pro-inflammatory cytokines in serum and colon, while exhibiting a positive correlation with pain threshold (Fig. 2J). The finding indicated a correlation between a deficiency in butyric acid-producing bacteria and elevated uric acid levels, which may contribute to the progression of gouty arthritis. Given the established role of butyric acid in maintaining intestinal homeostasis and barrier function, we proceeded to evaluate the extent of inflammatory cell infiltration and expression of TJ protein expression in *Uox*-KO mice through HE staining and immunohistochemistry, respectively. Significant inflammatory cell infiltration (Fig. 2K) and decreased expression of the TJ proteins occludin and ZO-1 (Fig. 2L–M) were observed in the intestine of *Uox*-KO mice.

### Administration of *C. Butyricum* and butyrate attenuated macrophage polarization and gouty arthritis

**Fig. 3:**
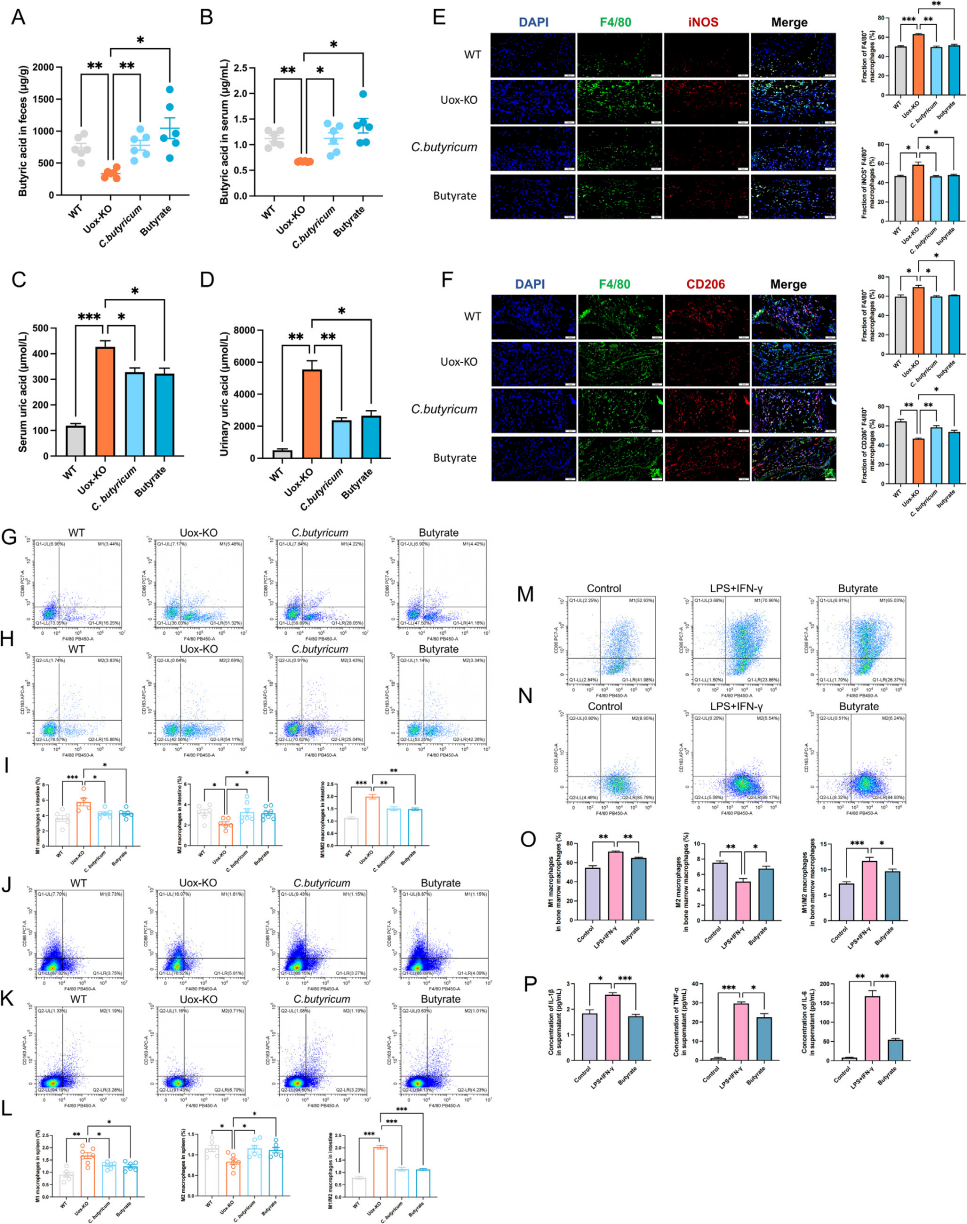
**Effects of *C. butyricum* and butyrate supplementation on the macrophage polarization and gouty arthritis in *Uox*-KO mice.** (A–B) Serum and faecal butyric acid concentrations after *C. butyricum* and butyrate treatment. (C–D) The level of serum uric acid and urinary uric acid after *C. butyricum* and butyrate treatment. (E–F) Immunofluorescence staining of F4/80, iNOS, and CD206 on mouse footpad. (G–L) Representative flow cytometry plots of the intestinal and splenic M1 and M2 macrophage populations, and percentage of M1 macrophage cells, M2 macrophage cells, and M1/M2 ratio after *C. butyricum* and butyrate treatment. (M–O) Representative flow cytometry plots of M1 and M2 macrophage populations, and percentage of M1 macrophage cells, M2 macrophage cells, and M1/M2 ratio in LPS-induced BMDMs after butyrate treatment. (P) The concentrations of IL-1β, TNF-α, and IL-6 in BMDMs supernatant. Values are expressed as mean ± SEM. “ns” represents not significant; * *P* < 0.05; ** *P* < 0.01; *** *P* < 0.001.

To clarify the role of butyric acid-producing bacteria and butyrate in the treatment of gouty arthritis, we administered the *C. butyricum,* a representative butyrate acid-producing bacterium whose primary metabolite is butyric acid (Fig. S2A), and butyrate to *Uox*-KO mice via gavage for 6 weeks. qPCR analysis revealed significantly reduced abundance of *C. butyricum* in *Uox*-KO mice (Fig. S2B). The findings demonstrated that both *C. butyricum* and butyrate were efficacious in elevating the levels of butyric acid in the intestinal tract and serum (Fig. 3A–B), and in reducing SUA and UUA levels in *Uox*-KO mice (Fig. 3C–D). Meanwhile, strong anti-inflammatory effects were observed after intervention with *C. butyricum* or butyrate, as evidenced by a reduction of inflammatory cell infiltration in the paw pads (Fig. S3A), a decrease in swelling (Fig. S3B), and an increase in pain threshold (Fig. S3C). Furthermore, the results demonstrated that the administration of *C. butyricum* and butyrate mitigated the inflammatory infiltration in the intestine and serum (Fig. S3D-I), and restored the expression of occludin and ZO-1 (Fig. S3J-L).

To assess the impact of intervention on macrophage phenotype, we performed immunofluorescence staining and observed a notable reduction in the number of iNOS^+^ cells in the mice treated with *C. butyricum* or butyrate. Furthermore, the proportion of CD206^+^ cells was markedly elevated (Fig. 3E–F). The similar changes, decreased M1 phenotypes and increased M2 macrophages, were observed in both intestine and spleen by flow cytometry analysis (Fig. 3G–L), indicating that *C. butyricum* and butyrate treatment modulates local and systemic macrophage polarization.

In *vitro* intervention of LPS-induced BMDMs provided further verification of the regulatory effects of butyrate on macrophage polarization and the inflammatory response. The working concentration of butyrate on BMDMs was determined through CCK-8 screening, with 1.25 mM butyrate selected for the subsequent experiments (Fig. S4). Consistent with the in *vivo* results, the flow cytometry results showed that butyrate intervention significantly suppressed M1 polarization and promoted M2 polarization (Fig. 3M–O). Meanwhile, it was confirmed by ELISA with the significant decline in the expression of IL-1β, IL-6, and TNF-α in the butyrate-treated BMDMs (Fig. 3P). The aforementioned results collectively indicated that *C. butyricum* and butyrate could effectively alleviate gouty arthritis and restore unbalanced macrophage polarization. The supplementation of *C. butyricum* and butyrate is a promising treatment for lowering uric acid and inhibiting inflammation.

### Aberrant intestinal miRNA profile correlated with dysregulated gut microbiota and macrophage polarization

**Fig. 4:**
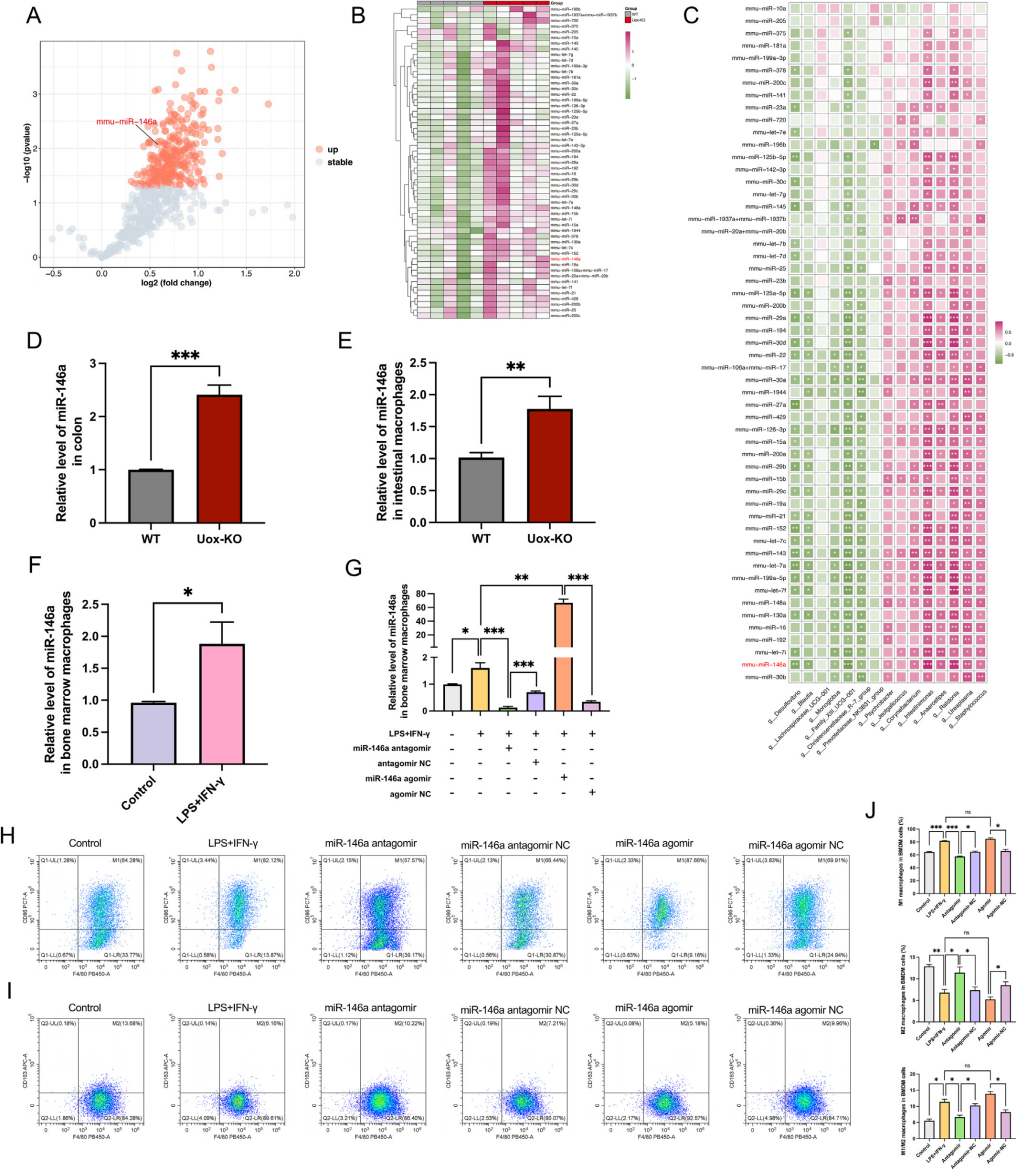
**Expression of miR-146a regulated macrophage polarization.** (A) The volcano plot of the colonic miRNA expression between WT and *Uox*-KO mice (n = 5 per group). Differentially expressed miRNA (*P* value < 0.05) is highlighted in orange. (B) Heatmap of selected 55 miRNAs in the colon of WT and *Uox*-KO mice. Data were normalized for heatmap visualization. (C) Spearman's rank correlation analysis was conducted between discrepant microbial taxa and 55 miRNAs. Positive correlations are displayed in red and negative correlations in green. The intensity of the colour is proportional to the correlation coefficient. * *P* < 0.05; ** *P* < 0.01; *** *P* < 0.001. (D) qRT-PCR of miR-146a expression in mice colon tissues. (E) qRT-PCR of miR-146a expression in mice primary intestinal macrophages. (F) qRT-PCR of miR-146a expression in LPS-induced BMDMs. (G) qRT-PCR of miR-146a expression in BMDMs transfected with miR-146a mimics. (H-J) Representative flow cytometry plots of M1 and M2 macrophage populations, and percentage of M1 macrophage cells, M2 macrophage cells, and M1/M2 ratio in BMDMs transfected with miR-146a mimics. Values are expressed as mean ± SEM. “ns” represents not significant; * *P* < 0.05; ** *P* < 0.01; *** *P* < 0.001.

Emerging studies have revealed that miRNAs are one of the intermediate factors linking the gut barrier and immune regulation. These molecules are capable of interacting with the gut microbiota in a reciprocal manner and have been shown to play a role in the pathogenesis of gout [24,25]. Therefore, we systematically investigated the changes and functions of intestinal miRNA in *Uox*-KO mice. In total, 599 colonic miRNAs were identified using the NanoString nCounter digital quantification platform. We found that the expression of a variety of miRNAs, including miR-146a, a specific microRNA that has been demonstrated to play a vital role in regulating inflammatory and innate immune responses and is associated with the development of gout [26], was significantly upregulated in the gut of *Uox*-KO mice (Fig. 4A–B).

To better investigate the correlation between gut flora and miRNA expression, 55 miRNAs with an average expression of over 1000 RPM were selected for further differential analysis. A correlation analysis was performed to reveal the characteristic miRNAs that were influenced by the disparate bacterial genera. The results demonstrated a negative correlation between miR-146a expression and the abundance of the majority of butyric acid-producing bacteria found in the study (Fig. 4C), suggesting a close interaction between miR-146a and these bacteria. Notably, correlation analysis between miRNAs and gout indices revealed that miR-146a exhibited the strongest positive association with SUA levels, further implicating its role in gout pathogenesis (Fig. S5). The increased miR-146a expression in colon tissues of *Uox*-KO mice was further validated by qRT-PCR method (Fig. 4D). In addition, elevated miR-146a expression was also detected in intestinal macrophages and LPS-induced BMDMs (Fig. 4E–F), indicating a role for miR-146a in macrophage polarization. To elucidate the impact of miR-146a on the polarization of macrophages, we up- and down-regulated miRNA-146a expression with agomiR-146a and antagomiR-146a, respectively, in BMDMs (Fig. 4G). The overexpression of miR-146a was observed to promote M1 polarization, whereas the suppression of miR-146a was seen to inhibit the M1 macrophage phenotype (Fig. 4H–J), proving that miR-146a participates in the modulation of macrophage polarization.

### miR-146a regulated macrophage polarization through SOCS7/JAK2-STAT3 signaling pathway

**Fig. 5:**
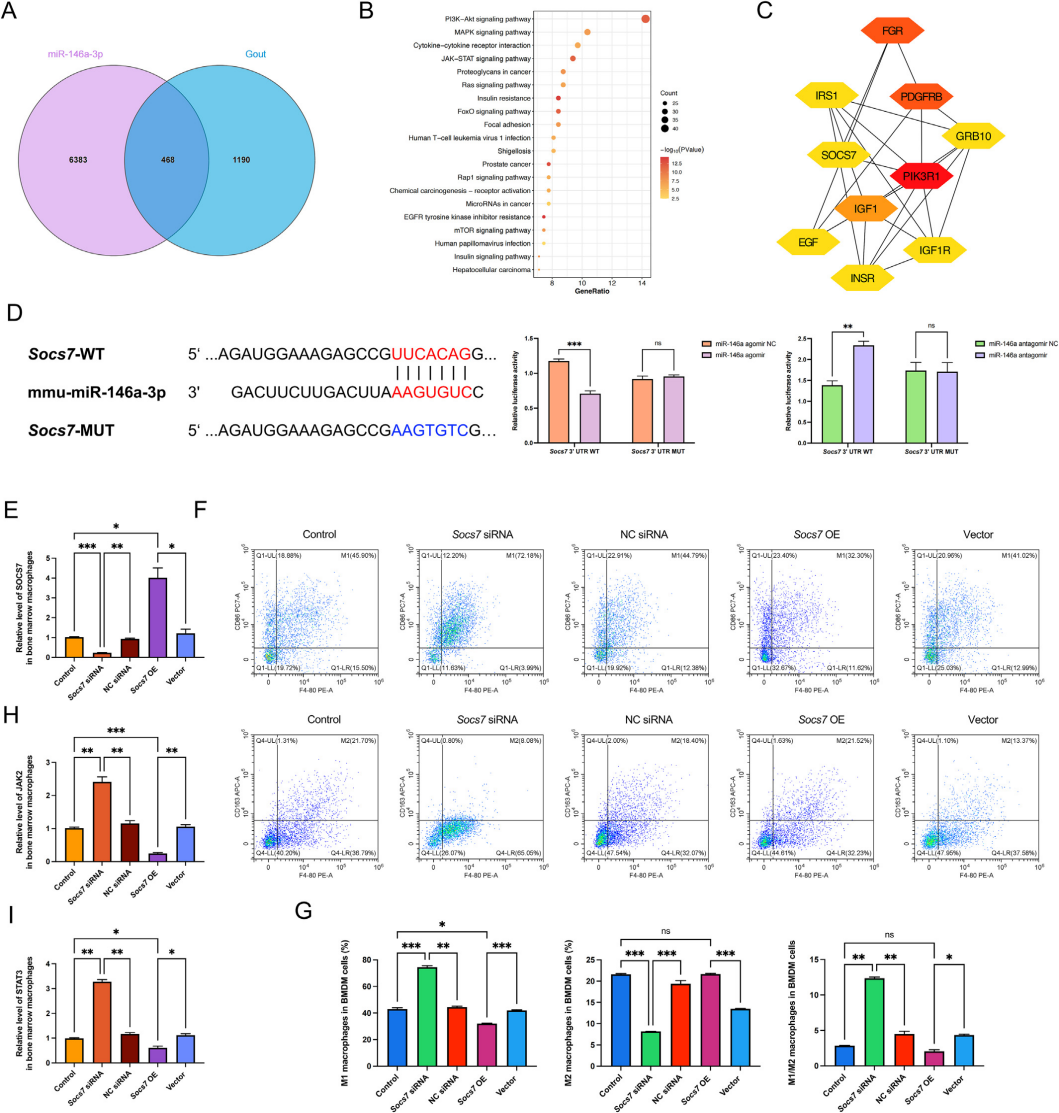
***Socs7* modulated macrophage polarization.** (A) Venn Diagram of the overlapping genes between the putative miR-146a-3p targets and gout-related genes. (B) Bubble plot of the top 20 KEGG-enriched pathways obtained from the overlapping genes of miR-146a targets and gout-related genes. (C) The top 10 hub genes were screened from the PPI network of 468 cross-targets using CytoHubba. (D) The binding site of miR-146a in the *Socs7* 3′-UTR and the validation of direct binding of miR-146a and *Socs7* by dual-luciferase reporter assay. (E) *Socs7* mRNA expression levels in BMDMs transfected with *Socs7* siRNA and *Socs7* overexpression plasmid (*Socs7* OE). (F-G) Representative flow cytometry plots and percentage of macrophages from *Socs7* siRNA and *Socs7* OE treatment showing M1 and M2 macrophage polarization. (H) *Jak2* mRNA expression levels in BMDMs transfected with *Socs7* siRNA and *Socs7* OE. (I) *Stat3* mRNA expression levels in BMDMs transfected with *Socs7* siRNA and *Socs7* OE. Values are expressed as mean ± SEM. “ns” represents not significant; * *P* < 0.05; ** *P* < 0.01; *** *P* < 0.001.

To deeply investigate the potential mechanism of miR-146a in gout pathogenesis, we conducted a comprehensive search for gout-related genes across multiple databases, including GeneCards, CTD, ClinVar, and DisGeNET databases. This approach led to the identification of 1,658 DEGs. Furthermore, 6,851 predicted targets of miR-146a were obtained through the Target Scan, DIANA, miRWalk, and miRDB databases. A total of 468 common genes were screened through the intersection of the miR-146a target genes and those associated with gout (Fig. 5A). KEGG pathway analysis revealed that the inflammatory pathways, including PI3K-Akt, MAPK, and JAK-STAT, were enriched in the overlapping target genes (Fig. 5B). Among these, the JAK/STAT pathway plays a prominent role in macrophage polarization [27]. We further constructed a PPI network of 468 DEGs, and ten hub genes (PIK3R1, PDGFRB, FGR, SOCS7, INSR, EGF, IGF1, IRS1, IGF1R, and GRB10) were selected with high degrees using CytoHubba software. Subsequently, the aforementioned genes were employed to construct a hub gene network comprising 10 nodes and 24 edges (Fig. 5C). Studies have highlighted the essential role of SOCS proteins in regulating both innate and adaptive immunity. They function as a molecular switch, orchestrating the polarization of macrophages through the JAK-STAT pathway [28,29]. We, therefore, postulated that miR-146a may induce the skewed M1 phenotype via the SOCS7/JAK2-STAT3 pathway to promote gouty arthritis.

To substantiate this speculation, we first checked the target binding of *Socs7* mRNA and miR-146a. Our analysis revealed that miR-146a-3p could bind to continuous 7 bases in the 3′-UTR of *Socs7*. Luciferase reporter assay further revealed that the overexpression of miR-146a resulted in a reduction in luciferase activity in wild-type *Socs7*, whereas this was not observed in mutant *Socs7*. Conversely, transfection of miR-146a antagomir, an inhibitor of miR-146a, led to an increase in luciferase activity of wild-type *Socs7*. The results confirmed the direct target binding between miR-146a and the wild-type *Socs7* (Fig. 5D). We further investigated the role of *Socs7* in macrophage polarization by knockdown and overexpression experiments (Fig. 5E). *Socs7* siRNA significantly promoted M1 polarization and upregulated *Jak*2 and *Stat3* mRNA levels. However, *Socs7* overexpression reversed these effects, decreasing M1 polarization and suppressing *Jak2*/*Stat3* expression (Fig. 5F–I). These findings supported that *Socs7* plays a critical role in modulating macrophage polarization through the JAK2-STAT3 pathway.

**Fig. 6:**
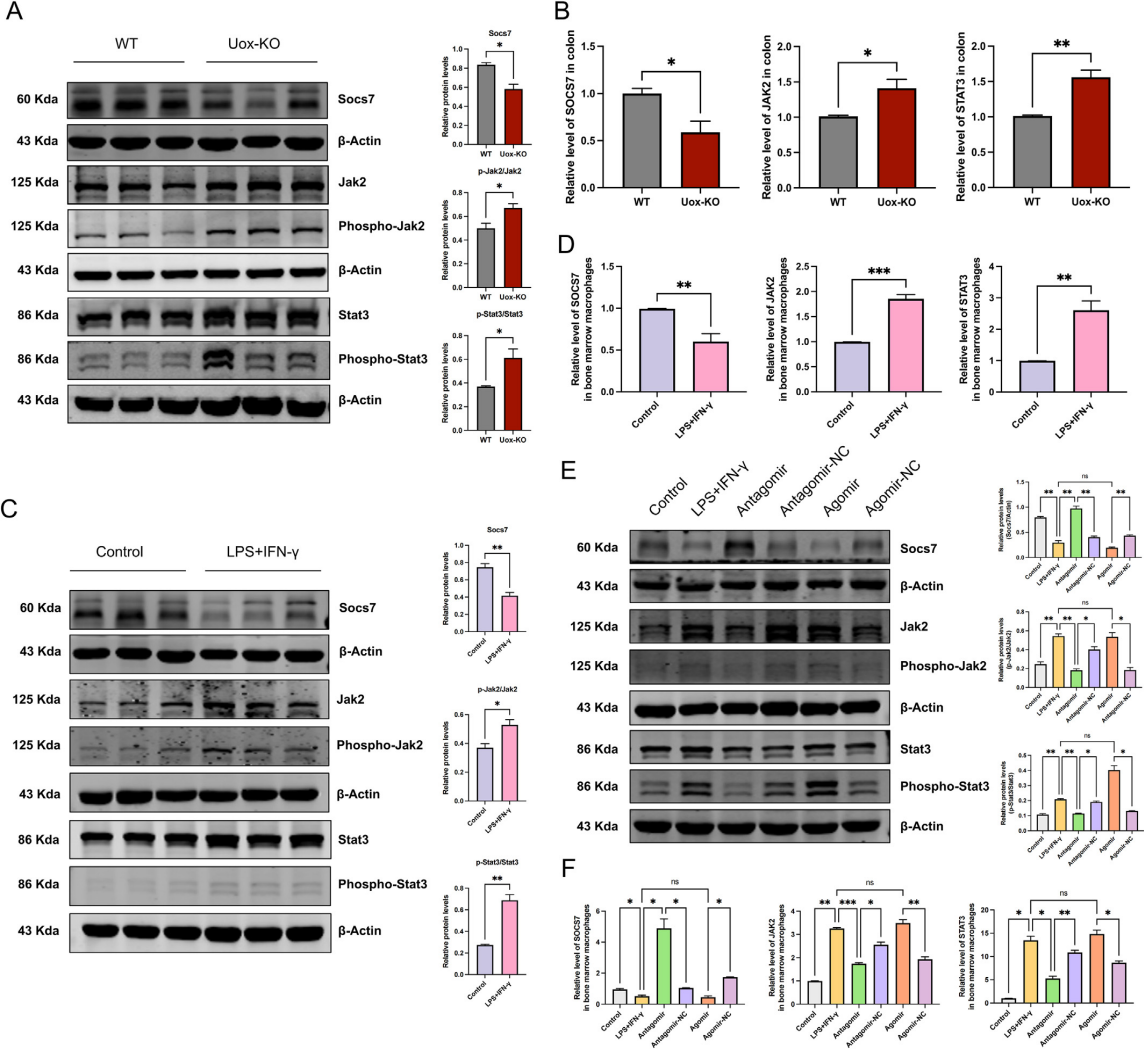
**miR-146a regulated macrophage polarization by regulating the SOCS7/JAK2-STAT3 signalling pathway.** (A-B) Protein and mRNA expression levels of SOCS7, JAK2, and STAT3 in the colon of WT and *Uox*-KO mice. (C-D) Protein and mRNA expression levels of SOCS7, JAK2, and STAT3 in BMDMs. (E-F) Protein and mRNA expression levels of SOCS7, JAK2, and STAT3 in BMDMs transfected with miR-146a mimics. Values are expressed as mean ± SEM. “ns” represents not significant; * *P* < 0.05; ** *P* < 0.01; *** *P* < 0.001.

To explore the molecular basis of these findings, we proceeded to examine the expression of SOCS7, JAK2, and STAT3 in the colon of *Uox*-KO mice. Western blot confirmed the significant reduction in SOCS7 levels and a notable increase in p-JAK2 and p-STAT3 levels (Fig. 6A). qRT-PCR revealed a notable decline in *Socs7* expression and an elevation in *Jak2* and *Stat3* expression in the *Uox*-KO mice (Fig. 6B). In addition, the results of the in *vivo* experiment were replicated in LPS-induced BMDMs, with the inhibition of SOCS7 and activation of JAK2 and STAT3 observed by both qPCR and western blot (Fig. 6C–D). The overexpression of miR-146a resulted in the suppression of SOCS7 expression and the promotion of JAK2 and STAT3 expression. Conversely, the inhibition of miR-146a exerted the opposite effect, thereby further verifying the regulatory effect of miR-146a on macrophage polarization via the SOCS7/JAK2-STAT3 pathway (Fig. 6E–F).

### *C. Butyricum* and butyrate alleviated gouty arthritis by suppressing the expression of miR-146a

**Fig. 7:**
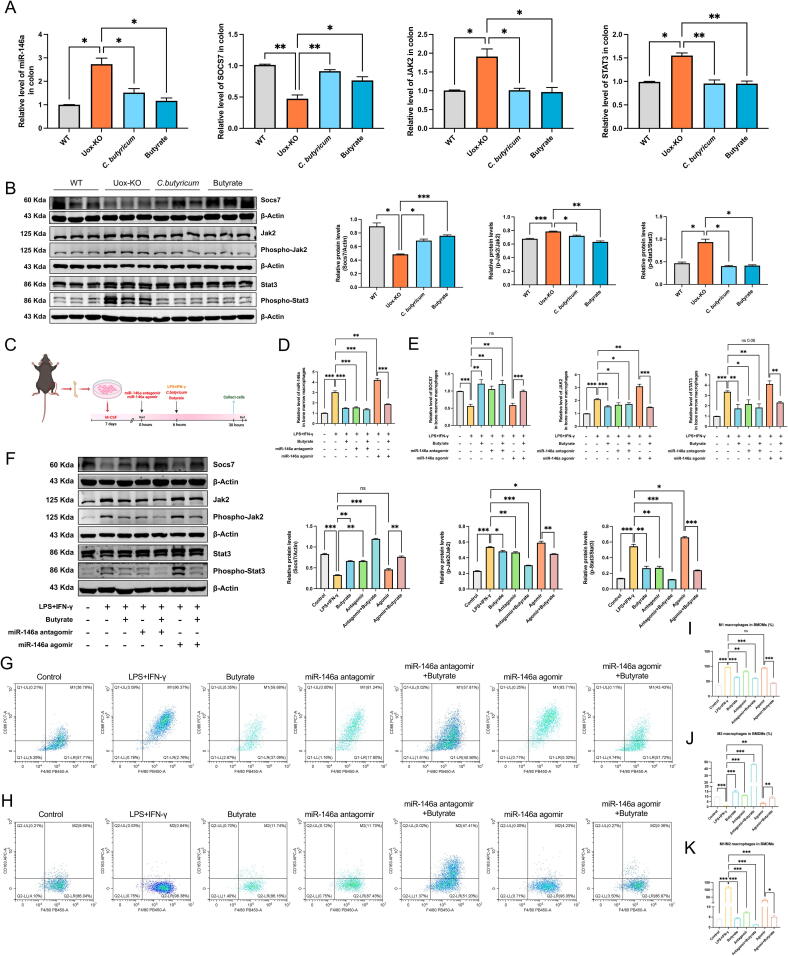
***C. butyricum* and butyrate alleviated gouty arthritis by regulating miR-146a expression.** (A-B) mRNA and protein expression levels of SOCS7, JAK2, and STAT3 in the colon after *C. butyricum* and butyrate treatment. (C) The experimental procedure of miR-146a transfection in BMDMs. (D) Changes of miR-146a expression in BMDMs transfected with miR-146a and butyrate treatment. (E-F) mRNA and protein expression levels of SOCS7, JAK2, and STAT3 in miR-146a-transfected or butyrated-treated BMDMs. (G-K) Representative flow cytometry plots showed M1 and M2 macrophage populations, and percentage of M1 macrophage cells, M2 macrophage cells, and M1/M2 ratio in miR-146a-transfected or butyrated-treated BMDMs. Values are expressed as mean ± SEM. “ns” represents not significant; * *P* < 0.05; ** *P* < 0.01; *** *P* < 0.001.

To elucidate the impact of *C. butyricum* on miR-146a, we assessed the levels of miR-146a and its downstream targets *Socs7*, *Jak2*, and *Stat3* in the intestine and spleen of treated *Uox*-KO mice. The results of qPCR showed that, in comparison to the *Uox*-KO group, *C. butyricum* and butyrate administration obviously attenuated the expression of miR-146a, concomitant with upregulated *Socs7* and suppressed *Jak2* and *Stat3* mRNA levels in intestinal tissues (Fig. 7A). Consistent results were observed in the mouse spleen (Fig. S6A). These results were validated by western blot with enhanced SOCS7, reduced p-JAK2 and p-STAT3 after *C. butyricum* and butyrate intervention (Fig. 7B).

In *vitro* results were analogous. Butyrate was observed to effectively suppress the expression of miR-146a in LPS-induced BMDMs, and to counteract the elevation of miR-146a that was induced by AgomiR-146a transfection (Fig. 7C–D). Similarly, both qPCR and western blot results showed that butyrate treatment restored SOCS7 expression in LPS-induced and miR-146a-overexpressing BMDMs, and reduced the expression of JAK2 and STAT3 (Fig. 7E–F). These results provide compelling evidence for the regulatory effect of butyrate on miR-146a expression and its target genes. Furthermore, the results of flow cytometry revealed that butyrate regulates macrophage polarization by interfering with the expression of miR-146a (Fig. 7G–K). The alterations in the contents of cytokines IL-1β, IL-6, and TNF-α in the butyrate-treated and miR-146a-interfered BMDMs also provided corroboration for our hypothesis (Fig. S6B).

To further characterize the regulatory role of miR-146a in gout-associated inflammation, we extended our investigation to MSU crystal-stimulated models. Mirroring LPS-induced effects, MSU exposure (250 µg/mL, 6 h) triggered a pronounced M1 macrophage polarization, accompanied with miR-146a upregulation and SOCS7 expression (Fig. S7A-C). Notably, butyrate treatment attenuated these changes, reducing miR-146a levels, restoring SOCS7 expression, inhibiting JAK2-STAT3 expression, and attenuating MSU crystal-induced M1 polarization (Fig. S7A-C). Taken together, the data presented collectively indicate that *C. butyricum* and its metabolite butyrate exert a beneficial effect on gouty inflammation by regulating macrophage miR-146a expression.

### Butyric-acid producing bacteria and miR-146a deficiency in gouty arthritis patients

**Fig. 8:**
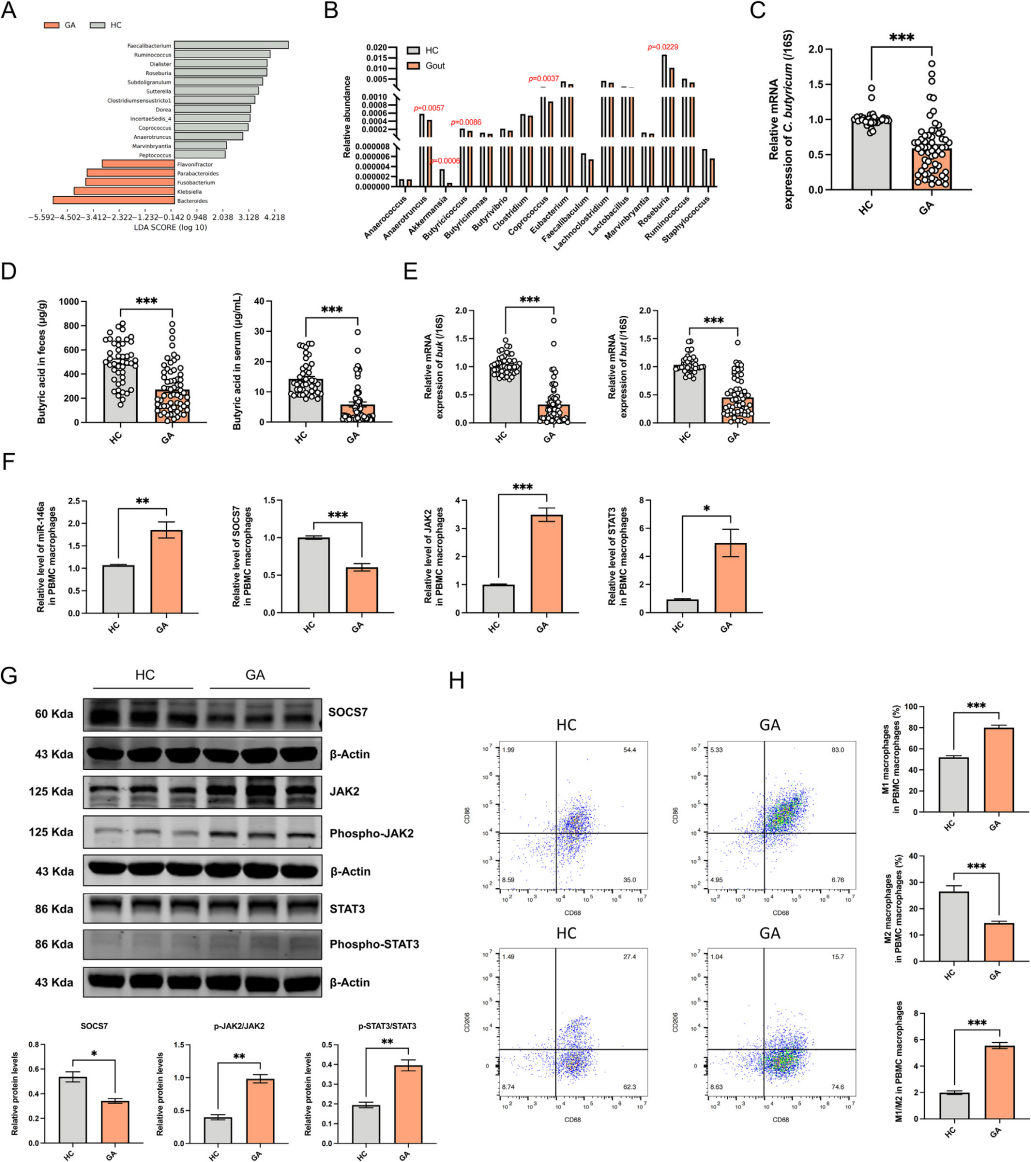
**Butyrate-producing bacteria and miR-146a dysregulation in gouty arthritis.** (A) The abundance of butyric acid-producing bacteria was decreased in gout patients compared to healthy controls by LEfSe analysis. (B) Relative abundance of butyric acid-producing bacteria in the public case-control metagenomic sequencing data. (C) Relative abundance of *C. butyricum* in healthy controls and gout patients (HC: n = 45; GA: n = 62). (D) Faecal and serum butyric acid concentrations in healthy controls and gout patients (HC: n = 45; GA: n = 62). (E) Relative expression of *Buk* and *But* in human intestinal contents (HC: n = 45; GA: n = 62). (F-G) qRT-PCR of miR-146a expression in human PBMC macrophages of HC and GA patients, and mRNA and protein expression levels of SOCS7, JAK2, and STAT3 in PBMC macrophages. (H) Representative flow cytometry plots showed M1 and M2 macrophage populations, and percentage of M1 macrophage cells, M2 macrophage cells, and M1/M2 ratio in human PBMC macrophages. Values are expressed as mean ± SEM. * *P* < 0.05; ** *P* < 0.01; *** *P* < 0.001.

In order to ascertain the clinical significance of our findings in *Uox*-KO mice, we conducted a reanalysis of our previous 16S rRNA amplicon sequencing data from gout patients [30]. In alignment with the observations made in the animal model, the population diagnosed with gout also exhibited a reduction in the abundance of butyric acid-producing bacteria (Fig. 8A). We further retrieved the public case-control metagenomic sequencing data set, which contains 221 gout patients and 86 healthy controls (accession number: CNP0000284), and revealed a decline in the prevalence of butyric acid-producing bacteria (Fig. 8B, Table S6). Species-specific qPCR confirmed the depletion of *C. butyricum* in gout patients (Fig. 8C). Consistent findings across independent trials suggest a potential link between the reduced abundance of butyric-acid bacteria and the development of gout. Moreover, GC–MS analysis demonstrated a significant decrease in butyrate levels in both fecal and serum samples from the patient cohort (Fig. 8D). qPCR analysis supported the diminution in the expression of butyrate metabolic enzymes *Buk* and *But* (Fig. 8E). Subsequently, PBMCs were harvested from the patients and healthy controls, and induced to differentiate into macrophages. Significantly elevated expression of miR-146a was observed in patients with gouty arthritis, along with decreased SOCS7 expression and increased JAK2 and STAT3 expression levels (Fig. 8F–G). Flow cytometry substantiated the imbalance of macrophage differentiation in the peripheral blood of patients with gouty arthritis, exhibiting an increase in M1 macrophages and a concomitant decrease in M2 macrophages (Fig. 8H). These findings further indicated that a deficiency in butyric acid-producing bacteria, a reduction in butyrate content, and an abnormal elevation of miR-146a are critical for the development of gout, leading to an imbalance in macrophage differentiation.

## Discussion

**Fig. 9:**
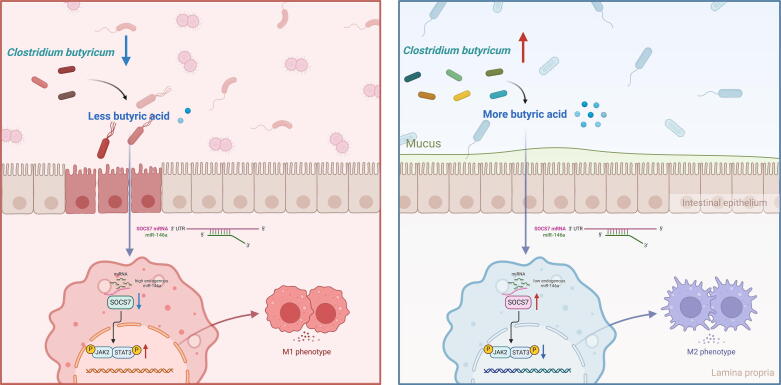
**Schematic schema of *C. butyricum*-mediated immunomodulation in gouty arthritis.** This schematic summarized the potential mechanism through which *C. butyricum* and its metabolic product butyrate influence macrophage polarization dynamics in gouty arthritis. In gouty conditions (left panel), depletion of butyrate-producing bacteria leads to systemic butyrate deficiency. This metabolic perturbation upregulates miR-146a expression, which directly suppresses SOCS7 transcription. The SOCS7 downregulation activates JAK2-STAT3 signalling pathway, driving predominant M1 macrophage polarization and exacerbating inflammation. *C. butyricum* supplementation (right panel) restores butyrate production, which normalizes miR-146a expression, thereby releasing SOCS7 suppression and shifting macrophage polarization toward the M2 phenotype. This immunophenotypic shift resolves inflammatory responses and ameliorates gouty inflammation. This image was created with BioRender (https://biorender.com/).

There is mounting evidence that the intestinal microbiome is critical for the development and progression of gout [31,32]. Variations in the structure and metabolism of the gut microbiota led to the abnormalities in uric acid degradation and generation, the increased release of pro-inflammatory mediators, and the damage to the intestinal barrier. Although prospective therapeutic approaches with a focus on gut microbiota for the prevention and treatment of gout have been highlighted, the study that explores the role of a single strain and its specific molecular mechanism in gouty arthritis is limited. This study is the first to demonstrate the beneficial role of *C. butyricum,* a butyrate-producing bacterium, and its metabolite in the treatment of gouty arthritis, providing a promising way to alleviate gouty inflammation (Fig. 9).

Research conducted by our group and others have previously demonstrated significant depletion of butyrate-producing bacteria, including *Bifidobacterium, Butyricicoccus, Faecalibacterium prausnitzii,* and *Oscillibacter,* in both gout patients and experimental animal models [19,33,34]. Our current findings corroborate this microbial dysbiosis pattern in *Uox*-KO mice and patients with gouty arthritis*.* Importantly, longitudinal intervention studies have established the causal relationship between butyrate-producer deficiency and hyperuricemia and gout pathogenesis [19]. Cohousing WT mice with *Uox*-KO counterparts for 8 weeks induced *C. butyricum* depletion and serum uric acid elevation, while fecal microbiota transplantation from gout-prone *Uox*-KO mice to WT recipients recapitulated butyrate deficiency and hyperuricemia within 4 weeks (Fig. S8A-D). In the present study, the significant reduction in *C. butyricum* abundance, the downregulation of butyrate metabolic enzymes (*Buk* and *But*), and the decreased butyrate content in both serum and feces were observed across species, suggesting the fundamental role of butyrate deficiency in gout pathogenesis. What’s more, Spearman's correlation analysis revealed significant inverse correlations between the abundance of the butyrogenic taxa and gout indicators. These findings position butyrate-producing bacteria and their metabolic products as potential biomarkers and therapeutic targets in gout management. Despite extensive research on the roles of butyrate-producing bacteria and butyrate in the treatment of various diseases [35,36], the effects of this bacterial group and their metabolites on gouty arthritis remain poorly understood.

*C. butyricum*, a butyrogenic intestinal symbiont, has been the subject of investigation into its potential ameliorative or protective effects in a variety of diseases, including enteric infection [37], intestinal injury [38,39], metabolic disease [40], inflammatory bowel disease [41], cancer [35], and neurodegenerative disease [42]. The safety of probiotic *C. butyricum* has been well established [34]. The mechanisms of beneficial action of *C. butyricum* are involved in multiple pathways, including the modulation of the composition of the gut microbiome [43], the strengthening of the gut barrier [44], and the restoration of immune homeostasis by promoting Treg responses and suppressing Th1- and Th17-mediated inflammation [45,46]. Our study provides novel mechanistic insights by demonstrating that *C. butyricum*-derived butyrate modulates macrophage polarization through the miR-146a/SOCS7 axis.

Macrophagesare the most recognized and best characterized innate immune cells that mediates the entire process of gout. The polarization state of macrophage is a critical determinant of the resolution or progression of gouty inflammation. The classical M1-like and alternative M2-like phenotypes of macrophages can be modulated by the presence of various bacterial metabolites, including bile acids, SCFAs, amino acids, and polyamines [47,48]. The effects of butyrate, one of the most important and abundant SCFAs, on M2 polarization remain inconclusive, particularly in different contexts [49,50]. Although previous studies have indicated that butyrate may inhibit M2 polarization and asthma-related airway inflammation [50,51], it is typically defined as a histone deacetylase inhibitor [49,52,53] or G protein-coupled receptor activator [54] to promote M2 polarization and to abrogate inflammation. The current findings offer a novel perspective on the regulation of macrophage polarization through the targeting of miR-146a expression.

Over the past few decades, several studies have illuminated the impact of miRNAs in the pathogenesis of gout [55]. It has been documented that circulating microRNAs play a vital role in innate immune regulation and inflammatory response in gout through multiple immune response signaling pathways [[55], [56], [57]]. Although several miRNAs (e.g., miR-155 exacerbating inflammation via SHIP-1 suppression [58], miR-192-5p attenuating gouty arthritis through EREG inhibition [59]) have been implicated in gout-related inflammatory and metabolic processes, our study mainly focused on the role of miR-146a in gout pathogenesis. Multi-level validation revealed significant miR-146a upregulation across experimental models (*Uox*-KO mice, LPS- and MSU-stimulated BMDMs) and clinical specimens (PBMCs from gouty arthritis patients). Furthermore, its expression exhibited the strongest correlation with SUA, further highlighting its pathogenic significance. Consistent with our results, Dalbeth also detected the markedly elevated miR-146a expression in PBMCs from intercortical gout patients [8,13]. Zhang observed an enhanced expression of miR-146a in MSU-stimulated mouse BMDMs [60]. These findings indicate the conserved role for miR-146a in gout progression. Indeed, the mechanistic importance of miR-146a in macrophage polarization has been the subject of considerable investigation. Targeted delivery of miR-146a to macrophages has been employed as a means to mitigate inflammatory diseases such as acute respiratory distress syndrome [61]. Zhang et al. have revealed its capacity to drive M2 macrophage polarization via TRAF6 suppression in diabetic nephropathy [62]. Building on these foundations, our investigation uncovered a negative correlation between the expression of miR-146a and the severity of gouty arthritis. Through integrated bioinformatics prediction and functional validation, we elucidated that miR-146a orchestrates M2 macrophage polarization by targeting the SOCS7/JAK2-STAT3 pathway. A better understanding of the role of miRNAs, especially miR-146a, in the modulation of macrophage immunoregulation will help to clarify the complexity and the heterogeneity of gout pathogenesis.

Despite the paucity of research examining the interaction between *C. butyricum* and microRNAs, an increasing array of evidence suggests that miRNAs interact with the gut microbiota in a reciprocal manner, exerting a profound effect on the health status of the host [[63], [64], [65]]. Given that probiotics are regulators of miRNAs, it can be reasonably inferred that these molecules significantly contribute to the molecular mechanisms underlying how probiotics maintain body homeostasis. The immunomodulatory potential of this intricate interplay between bacteria, host cells, and miRNAs has emerged as a novel therapeutic approach in ankylosing spondylitis [65,66]. In the context of gout, the administration of *C. butyricum* and butyrate has been demonstrated to significantly suppress the expression of miR-146a, thereby regulating the phenotypes of macrophages and alleviating the severity of gout arthritis, supporting the assertion that this novel approach is also effective in the treatment of gout.

There are several limitations in our study as well. First, further elucidation is required regarding the mechanism of SOCS7/JAK2-STAT3 signaling in macrophage polarization. Secondly, the direct observation of clinical intervention effects of *C. butyricum* and butyrate should be further investigated in the application of clinical trials. Future exploration of these issues may further elucidate the relationship between microflora and miRNA, and provide a potential direction and strategy for the clinical management of gout.

In conclusion, we have systematically investigated the role and mechanism of gut microbiota dysbiosis, with a particular focus on the reduction of butyrate-producing bacteria, in macrophage polarization and gout pathogenesis in *vivo* and in *vitro*. Our findings highlight *C. butyricum* and butyrate as promising therapeutic candidates for the treatment of gouty arthritis. A novel potential treatment approach has been explored to regulate macrophage polarization and inflammatory response through miR-146a and the SOCS7/JAK2-STAT3 signaling pathway. The findings of our study provide a theoretical basis for the targeting of intestinal flora in the treatment of gout.

## Conclusion

This study systematically investigated the role and mechanism of intestinal flora perturbation, especially the reduction of butyrate-producing bacteria and the resulting decline in local and systemic butyric acid levels, in macrophage polarization and gout pathogenesis in *vivo* and in *vitro*. The role of *C*. *butyricum* and its metabolite in regulating the SOCS7/JAK2-STAT3 pathway through miR-146a, thereby affecting the polarization of macrophages and inflammatory response, is explained, providing a theoretical basis for targeting intestinal flora in the treatment of gout.

## Data availability statement

The raw sequencing data from this study have been submitted to the National Center for Biotechnology Information (NCBI) Sequence Read Archive (SRA) database. The submission is identified by the BioProject accession number PRJNA1104750. The clinical data set generated for the current study is publicly available from the SRA under accession number PRJNA359624 and the metagenomic shotgun sequencing data have been deposited in the CNGB Nucleotide Sequence Archive (CNSA) under accession code CNP0000284 [7]. Further data associated with this article may be obtained by contacting the corresponding authors.

## Animals

*Uox*-KO mice on the C57BL/6J background were provided by Shanghai Southern Model Biotechnology Co., Ltd. The mice were housed under standard environmental conditions in accordance with requirements of the Institutional Animal Care and Use Committee and this study was granted permission by the Laboratory Animal Management and Welfare Ethical Review Committee of Zhejiang Chinese Medical University (approval number: IACUC-20210621-16).

## Donor cohorts

Prior to their participation, an informed consent form was signed by all participants. The study population exclusively comprised males aged between 25 and 47 years. Confirmed healthy (n = 45) and gouty arthritis (n = 62) were enrolled for the butyric acid concentration study and the expression of *Buk* and *But*. Five subjects were selected from each group for the isolation of peripheral blood mononuclear cells (PBMCs). Prior to their participation, all participants signed an informed consent form for the management of personal anamnestic data, blood, and faecal samples. The study was approved by the Ethics Committee of Zhejiang Chinese Medical University (approval no. 20240329-6) and conducted in accordance with the ethical principles of the Declaration of Helsinki.

## Credit author statement

Siyue Song: investigation, validation, original draft, writing and revision; Kaiyue Shi: methodology, investigation, data analysis, and validation; Moqi Fan: investigation and data analysis; Xianghui Wen: experimental operation; Jiatao Li, Yining Guo, Yu Lou, Fusen Chen: animal husbandry and experimental operation; Jialu Wang: data analysis; Lin Huang: clinical specimen preparation; Chengping Wen: experimental guidance, manuscript revision, and supervision; Tiejuan Shao: experimental design, project management, and manuscript revision. All data are in-house data. All authors attest to the veracity and accuracy of the work.

## Declaration of competing interest

The authors declare that they have no known competing financial interests or personal relationships that could have appeared to influence the work reported in this paper.
