# Supplementary material for: Clostridium butyricum and its metabolites regulate macrophage polarization through miR-146a to antagonize gouty arthritis
Source: J Adv Res. 2025 May 19;80:943–60. doi: 10.1016/j.jare.2025.05.036 (PMC12869238; doi:10.1016/j.jare.2025.05.036)
Supplement: Supplementary Data 1 [file mmc1.docx]

**SUPPLEMENTARY DATA**

*Clostridium butyricum* and its metabolites regulate macrophage polarization through miR-146a to antagonize gouty arthritis

**Siyue Song^a, 1^, Kaiyue Shi^a, 1^**, Moqi Fan^a^, Xianghui Wen^a^, Jiatao Li^a^, Yining Guo^a^, Yu Lou^a^, Fusen Chen^a^, Jialu Wang^c^, Lin Huang**^a, b^**, **Chengping Wen****^a, b, *^, Tiejuan Shao^a,b, *^**

^a^ College of Basic Medical Sciences, Zhejiang Chinese Medical University, Hangzhou, 310053, China.

^b^ Center for Innovative Basic Research in Autoimmune Diseases in Medicine, Hangzhou, 310053, China.

^c^ The Second Clinical Medical College, Zhejiang Chinese Medical University, Hangzhou, 310053, China.

**^1^ These authors have contributed equally to this work.**

**^*^ Corresponding authors:** Prof. Chengping Wen, Email: [wengcp@163.com](mailto:wengcp@163.com)

Or Prof. Tiejuan Shao, [tiejuanshao@zcmu.edu.cn](mailto:tiejuanshao@zcmu.edu.cn)

E-mail addresses: songsiyue@126.com (S. Song), [sky20012001@qq.com](mailto:sky20012001@qq.com) (K. Shi), moq_921@163.com (M. Fan), [xhw6180@163.com](mailto:xhw6180@163.com) (X. Wen), [1210812074@qq.com](mailto:1210812074@qq.com) (J. Li), [Gyining99@163.com](mailto:Gyining99@163.com) (Y. Guo), [qq931069343@gmail.com](mailto:qq931069343@gmail.com) (Y. Lou), [2641214515@qq.com](mailto:2641214515@qq.com) (F. Chen), 2264800914@qq.com (J. Wang), [huanglin@zcmu.edu.cn](mailto:huanglin@zcmu.edu.cn) (L. Huang), wengcp@163.com (C. Wen), tiejuanshao@zcmu.edu.cn (T. Shao).

**Table S1. Primary antibodies used for IHC and IF analysis**

| Antibodies | Dilution | Source | Cat# |
| --- | --- | --- | --- |
| ZO-1 | 1:150 | Affinity | #AF5145 |
| Occludin | 1:150 | Affinity | #DF7504 |
| F4/80 | 10 μg/mL | Abcam | #90247 |
| iNOS | 1:100 | Abcam | #115819 |
| CD206 | 1:100 | Abcam | #64693 |
| DAPI | 1:1 | Absin | #9235 |

**Table S2. Antibodies used for flow cytometry analysis**

| Antibodies | Fluorophore | Source | Cat# |
| --- | --- | --- | --- |
| CD45 | BV605 | BD Pharmingen | #563053 |
| F4/80 | BV421 | BD Pharmingen | #565411 |
| CD86 | PE-Cy7 | BD Pharmingen | #560582 |
| CD163 | APC | Thermo Fisher Scientific | #17-1631-82 |
| Fixable Viability Stain 700 | APC700 | BD Pharmingen | #564997 |
| CD45 | BV605 | BD Pharmingen | # 560779 |
| CD68 | BV421 | BD Pharmingen | # 564943 |
| CD86 | PE-Cy7 | BD Pharmingen | # 561128 |
| CD206 | APC | BD Pharmingen | # 561763 |

**Table S3. Sequences of miR-146 mimics and siRNA experiment**

| miRNA name | Sense（5'-3'） | Anti-sense（5'-3'） |
| --- | --- | --- |
| miR 146a antagomir | AACCCAUGGAAUUCAGUUCUCA | - |
| NC antagomir | CAGUACUUUUGUGUAGUACAA | - |
| miR 146a agomir | UGAGAACUGAAUUCCAUGGGUU | CCCAUGGAAUUCAGUUCUCAUU |
| NC agomir | UUCUCCGAACGUGUCACGUTT | ACGUGACACGUUCGGAGAATT |
| *Socs7* siRNA | UGCCUACAUCUGUCCUUGUTT | ACAAGGACAGAUGUAGGCATT |
| NC siRNA | UUCUCCGAACGUGUCACGUTT | ACGUGACACGUUCGGAGAATT |

**Table S4. Primers for qRT-PCR analysis**

| Primers | Forward sequences | Reverse sequences |
| --- | --- | --- |
| Mmu-*Socs7* | TGACAAGAACTCAAAGTGCC | GGCTTCTTCTTGGAGGAGGA |
| Mmu-*Jak2* | TTGTGGTATTACGCCTGTGTATC | ATGCCTGGTTGACTCGTCTAT |
| Mmu-*Stat3* | CAATACCATTGACCTGCCGAT | GAGCGACTCAAACTGCCCT |
| Mmu-*β-actin* | GGCTGTATTCCCCTCCATCG | CCAGTTGGTAACAATGCCATGT |
| Mmu-*U6* | GCTTCGGCAGCACATATACTAAAAT | CGCTTCACGAATTTGCGTGTCAT |
| Mmu-*miR-146a* | CCTGAGAAGTGAATTCCATGGG | CTCAACTGGTGTCGTGGAGTC |
| Hsa-SOCS7 | ACAGGAAGGTTGGGATTCTC | CAGCACAGACTCTAACTCTG |
| Hsa-JAK2 | TGAGTTCGAAGCTAGCAGGG | AAGCCCGTCACAGTTGTCTC |
| Hsa-STAT3 | GGGAAGAATCACGCCTTCTAC | ATCTGCTGCTTCTCCGTCAC |
| Has-β-ACTIN | AGCGAGCATCCCCCAAAGTT | GGGCACGAAGGCTCATCATT |
| Hsa-U6 | ATTGGAACGATACAGAGAAGATT | GGAACGCTTCACGAATTTG |
| Hsa-miR-146a | ATTTTACAGGGCTGGGACAG | TCTTCCAAGCTCTTCAGCAG |
| *Buk* | TGCTGTWGTTGGWAGAGGYGGA | GCAACIGCYTTTTGATTTAATGCATGG |
| *But* | GCIGAICATTTCACITGGAAYWSITGGCAYATG | CCTGCCTTTGCAATRTCIACRAANGC |
| *Clostridium butyricum* | CGTGGGGAGCAAACAGGATT | CGCGAGGTTGCATCTCATTG |
| *16S* | GCAGGCCTAACACATGCAAGTC | CTGCTGCCTCCCGTAGGAGT |

**Table S5. Primary antibodies used for western blotting analysis**

| Antibodies | Dilution | Source | Cat# |
| --- | --- | --- | --- |
| Phospho-Jak2 (Tyr1007/1008) | 1:1000 | CST | #3771S |
| Jak2 (D2E12) | 1:1000 | CST | #3230S |
| Phospho-Stat3 (Tyr705) (D3A7) | 1:1000 | CST | #9145S |
| Stat3 (D3Z2G) | 1:1000 | CST | #12640S |
| Socs7 (E-8) | 1:1000 | Santa Cruz | #137241 |
| β-Actin (13E5) | 1:1000 | CST | #4970 |

**Table S6. Abundance of butyric acid-producing bacteria in HC and Gout groups**

| Genus | P value | Adjusted P value | Abundance mean in HC | Abundance mean in Gout patients | Abundance median in HC | Abundance median in Gout patients |
| --- | --- | --- | --- | --- | --- | --- |
| *Anaerococcus* | 7.92E-01 | 8.86E-01 | 0.000003601 | 0.000006611 | 0.000001502 | 0.000001477 |
| *Anaerotruncus* | 5.78E-03 | 2.66E-02 | 0.00071033 | 0.000482913 | 0.000582499 | 0.000436199 |
| *Akkermansia* | 6.31E-04 | 5.57E-03 | 0.000146402 | 0.00042533 | 0.000003499 | 0.000000764 |
| *Butyricicoccus* | 8.63E-03 | 3.55E-02 | 0.000280767 | 0.000227878 | 0.000222121 | 0.00016125 |
| *Butyricimonas* | 7.16E-01 | 8.39E-01 | 0.000150558 | 0.000150874 | 0.000119619 | 0.000087767 |
| *Butyrivibrio* | 2.21E-01 | 3.73E-01 | 0.000283643 | 0.000263686 | 0.000218995 | 0.000168046 |
| *Clostridium* | 1.76E-01 | 3.21E-01 | 0.000780605 | 0.000634725 | 0.000578219 | 0.000541258 |
| *Coprococcus* | 3.78E-02 | 1.05E-01 | 0.00170546 | 0.001438749 | 0.001272869 | 0.000887965 |
| *Eubacterium* | 1.86E-01 | 3.29E-01 | 0.006892417 | 0.007503134 | 0.003898301 | 0.002595078 |
| *Faecalibaculum* | 3.10E-01 | 4.73E-01 | 0.000010873 | 0.000012245 | 0.000006674 | 0.000005448 |
| *Lachnoclostridium* | 1.58E-01 | 2.94E-01 | 0.005005775 | 0.004883869 | 0.004182172 | 0.003276969 |
| *Lactobacillus* | 5.41E-01 | 6.82E-01 | 0.005915637 | 0.019992175 | 0.001510548 | 0.001122018 |
| *Marvinbryantia* | 6.66E-02 | 1.58E-01 | 0.000130709 | 0.000078615 | 0.00012152 | 0.000093435 |
| *Peptococcus* | 2.08E-01 | 3.59E-01 | 0.00000017 | 0.00000052 | 0 | 0 |
| *Roseburia* | 2.29E-02 | 7.62E-02 | 0.022556067 | 0.020703994 | 0.016581283 | 0.010365656 |
| *Ruminococcus* | 8.22E-02 | 1.85E-01 | 0.007737785 | 0.008323334 | 0.005160634 | 0.003436845 |


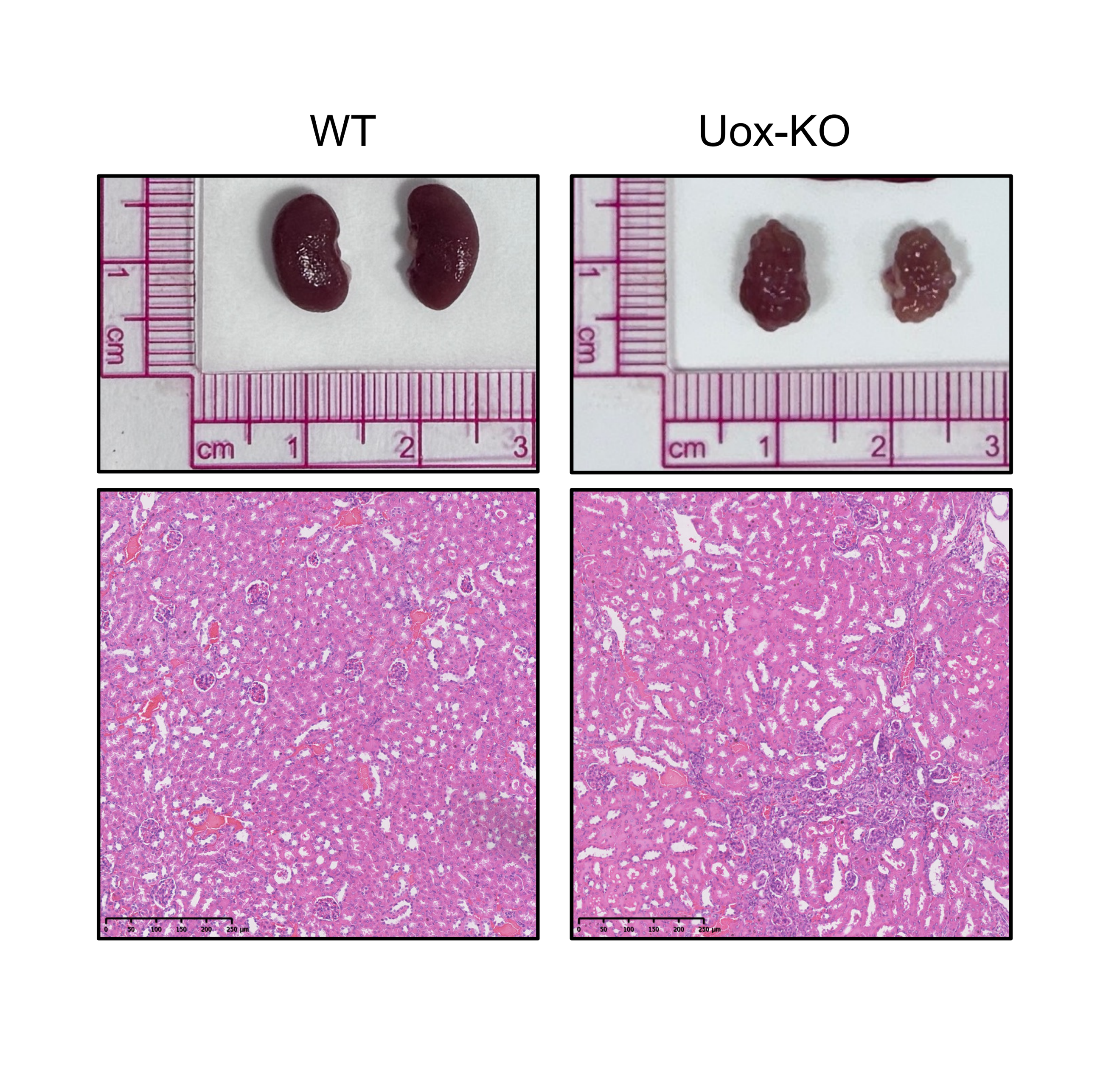


**Figure S1.** Kidney H&E staining. (Scale bar: 250 µm).

**
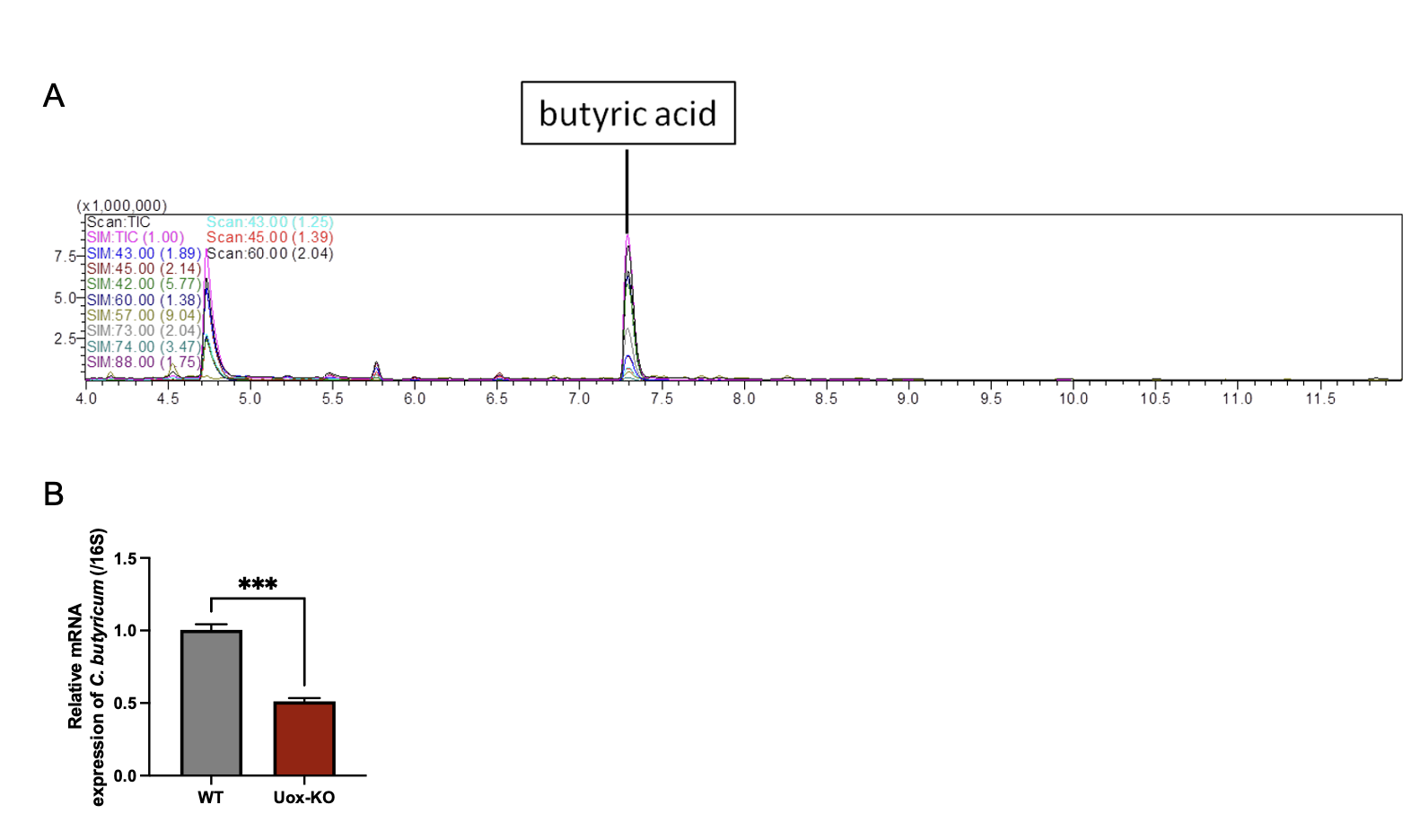
**

**Figure S2.** (A) GC/MS chromatograms of butyric acid, the major metabolite of *C. butyricum*. (B) Relative mRNA expression of *C. butyricum* in *Uox*-KO mice. Values are expressed as mean ± SEM. ****P* < 0.001.

**
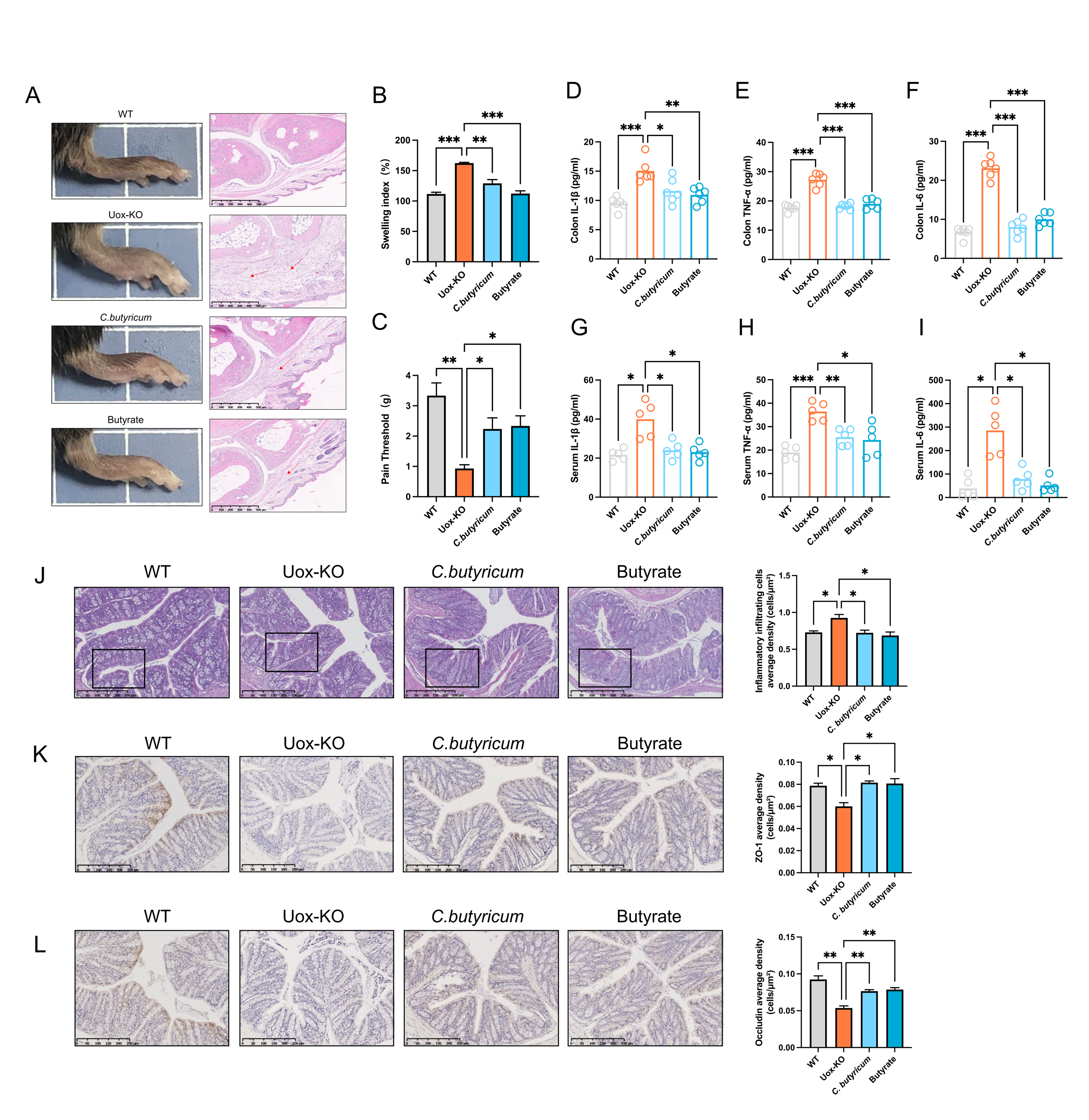
**

**Figure S3.** (A) Paraffin sections of mice claw were used for hematoxylin and eosin (H&E) staining with four groups mice. (Scale bar: 500 µm). (B) Footpad swelling. (C) Mechanical pain threshold. (D-I) The concentrations of IL-1β, TNF-α, and IL-6 in colon and serum after *C. butyricum* and butyrate treatment. (J) Colonic sections were stained with HE for histopathologic analysis (Scale bar: 250 µm). (K-L) Occludin and ZO-1 expression in the colon were measured by immunohistochemistry respectively (Scale bar: 250 µm). Values are expressed as mean ± SEM. “ns” represents not significant; **P* < 0.05, ***P* < 0.01, ****P* < 0.001.

**
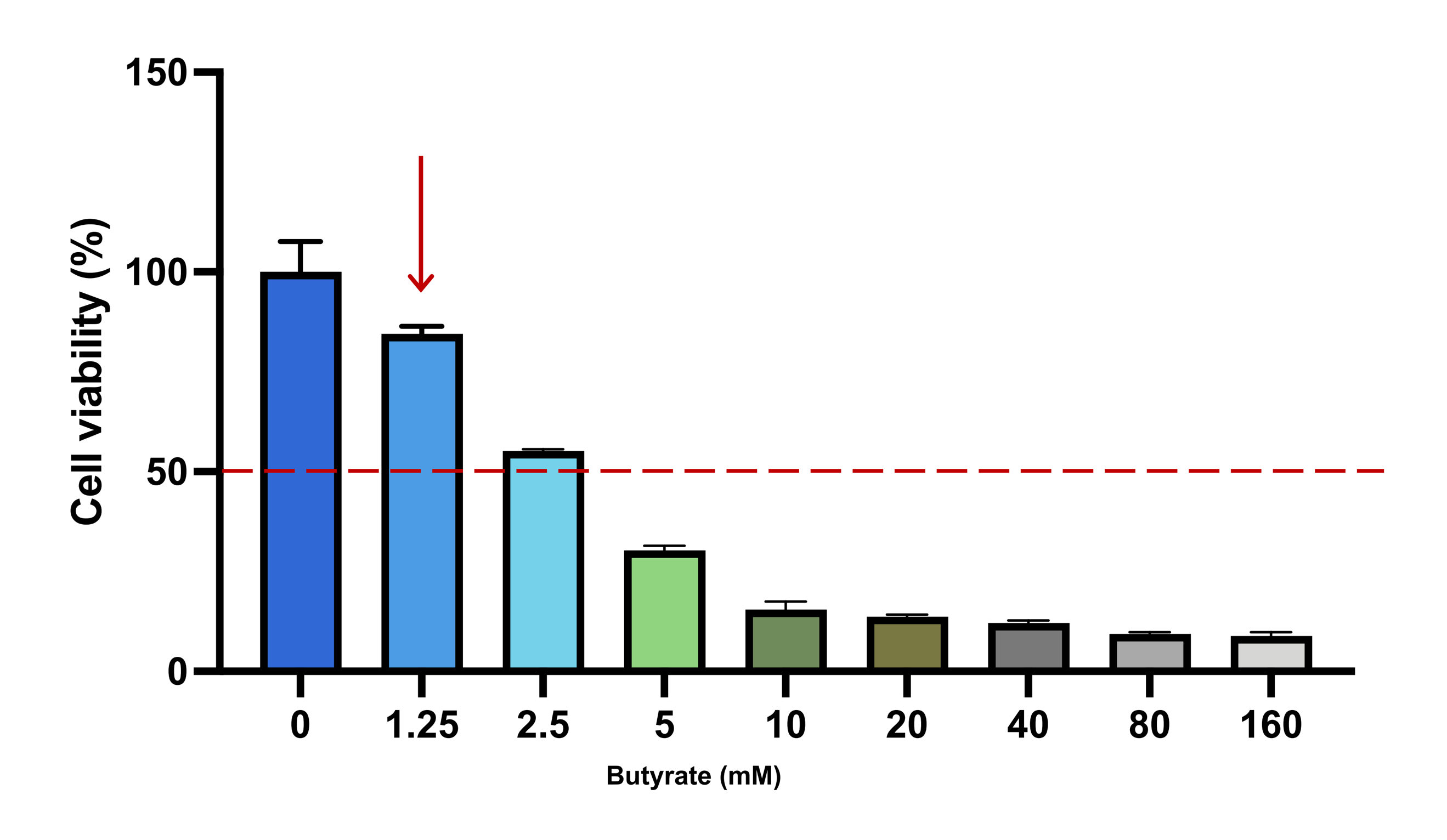
**

**Figure S4.** Cell viability of BMDMs with different concentrations of butyrate detected by CCK-8 assay.


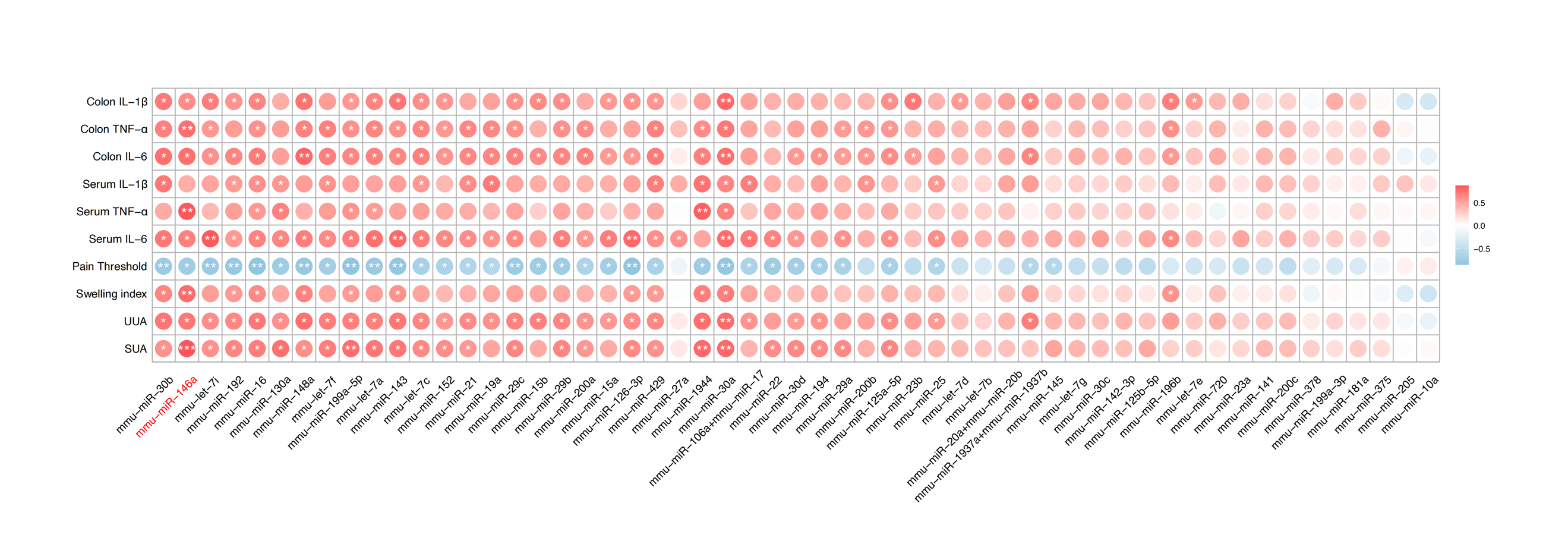


**Figure S5.** Spearman’s rank correlation analysis was conducted between gouty symptoms and 55 miRNAs. Positive correlations are displayed in red and negative correlations in blue. The intensity of the colour is proportional to the correlation coefficient. * *P* < 0.05; ** *P* < 0.01; *** *P* < 0.001.

**
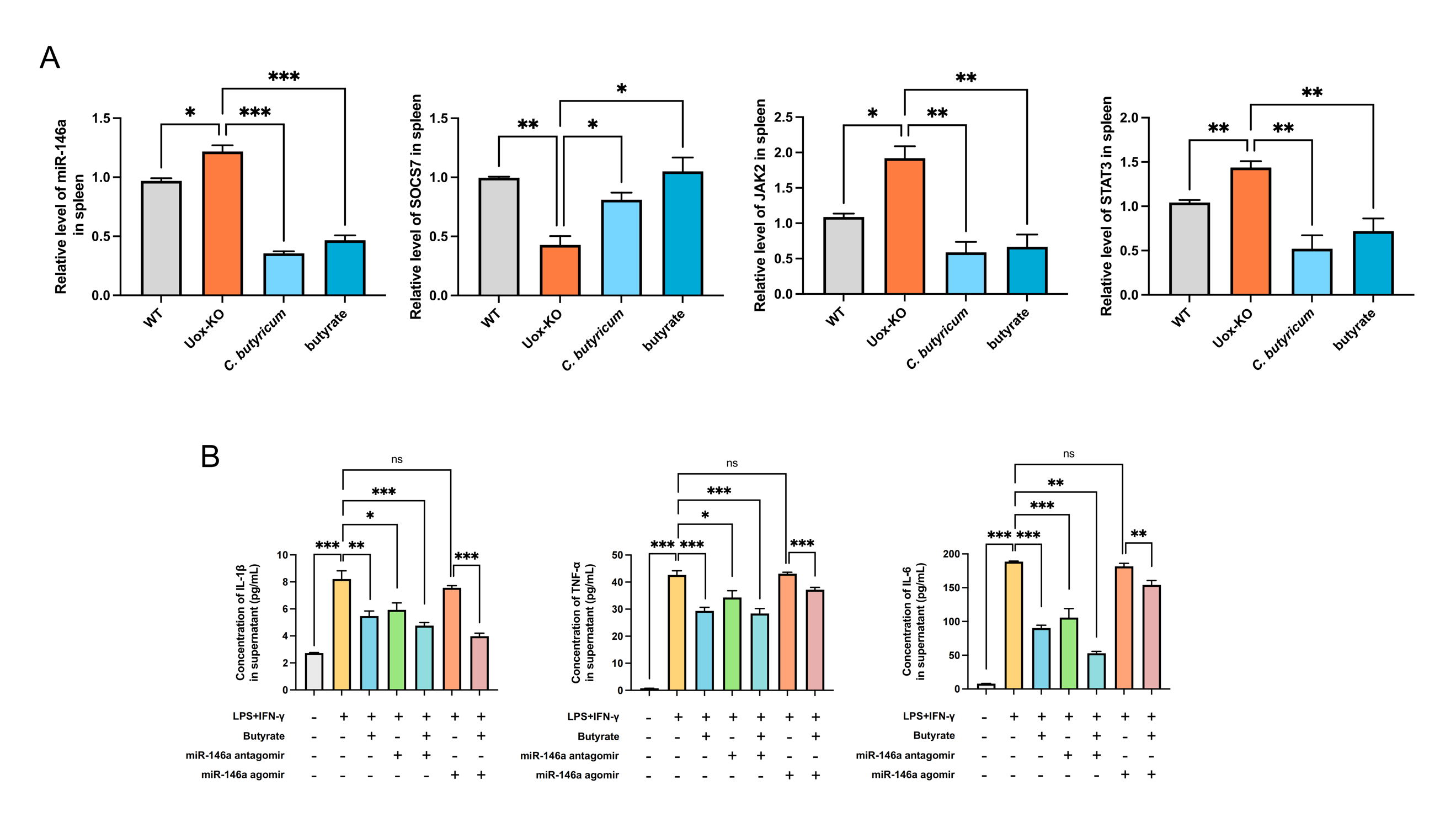
**

**Figure S6.** (A) mRNA expression levels of *Socs7*, *Jak*2, and *Stat3* in the spleen after *C. butyricum* and butyrate treatment by qPCR. (B) The concentrations of IL-1β, TNF-α and IL-6 in BMDMs supernatant after butyrate treatment were measured by Elisa. Values are expressed as mean ± SEM. “ns” represents not significant; **P* < 0.05, ***P* < 0.01, ****P* < 0.001.


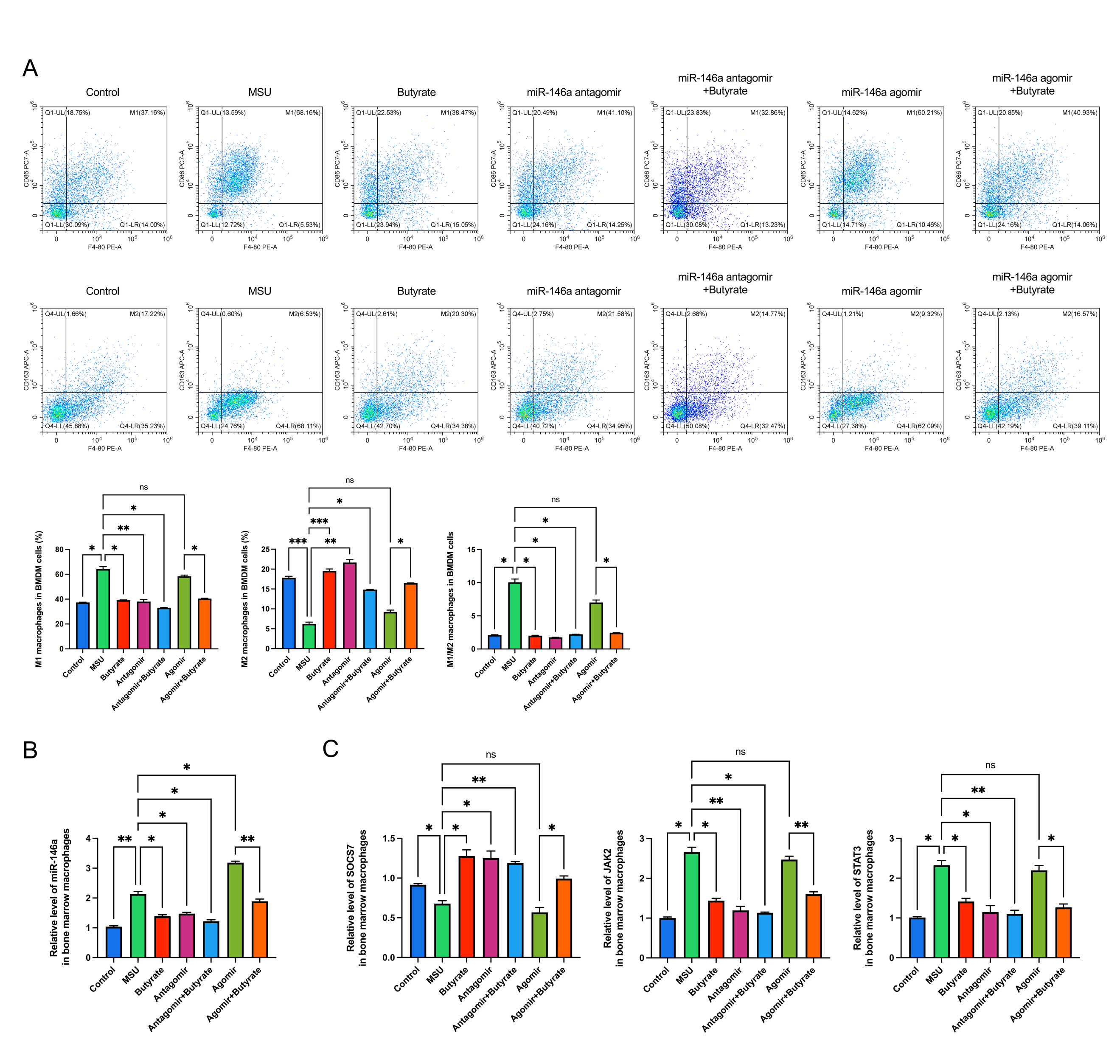


**Figure S7.** (A) Representative flow cytometry plots showed M1 and M2 macrophage populations, and percentage of M1 macrophage cells, M2 macrophage cells, and M1/M2 ratio in miR-146a-transfected or butyrated-treated BMDMs with MSU-stimulation. (B) Changes of miR-146a expression in BMDMs transfected with miR-146a and butyrate treatment with MSU-stimulation. (C) mRNA expression levels of *Socs7*, *Jak2*, and *Stat3* in miR-146a-transfected or butyrated-treated BMDMs. Values are expressed as mean ± SEM. “ns” represents not significant; **P* < 0.05, ***P* < 0.01, ****P* < 0.001.


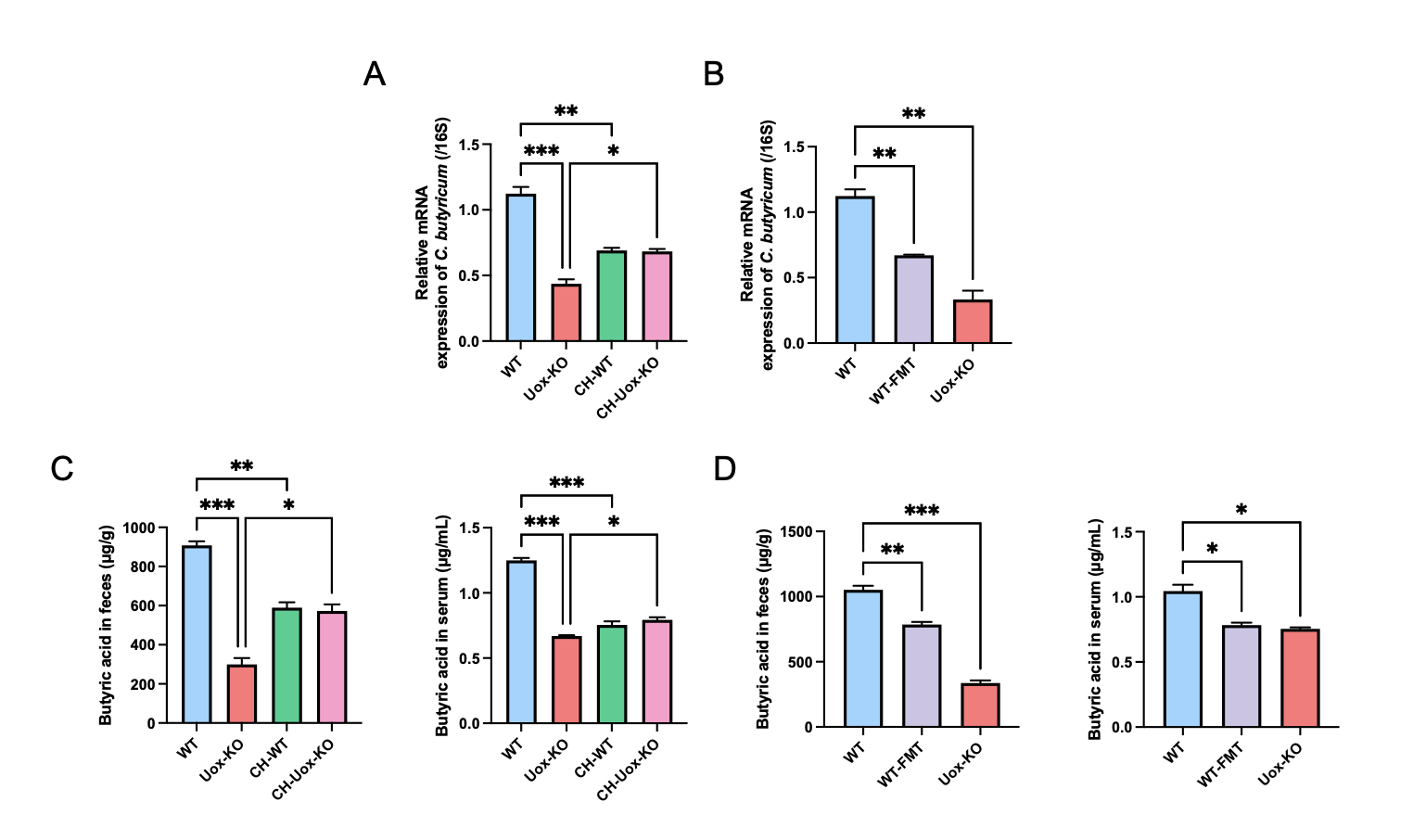


**Figure S8.** Relative mRNA expression of *C. butyricum* in faeces from co-housing mice and fecal microbiota transplantation (FMT) mice. For the co-housing experiment, WT and *Uox*-KO mice were housed together for 8 weeks after a 3-week adaptation period. For FMT, fresh faeces from *Uox*-KO mice were processed and orally administered to recipient mice for 4 weeks. (A-B) Relative mRNA expression of *C. butyricum* in fecal from co-housing mice and FMT mice. (C-D) Faecal and serum butyric acid concentrations in co-housing mice and FMT mice. Values are expressed as mean ± SEM. **P* < 0.05, ***P* < 0.01, ****P* < 0.001.

**
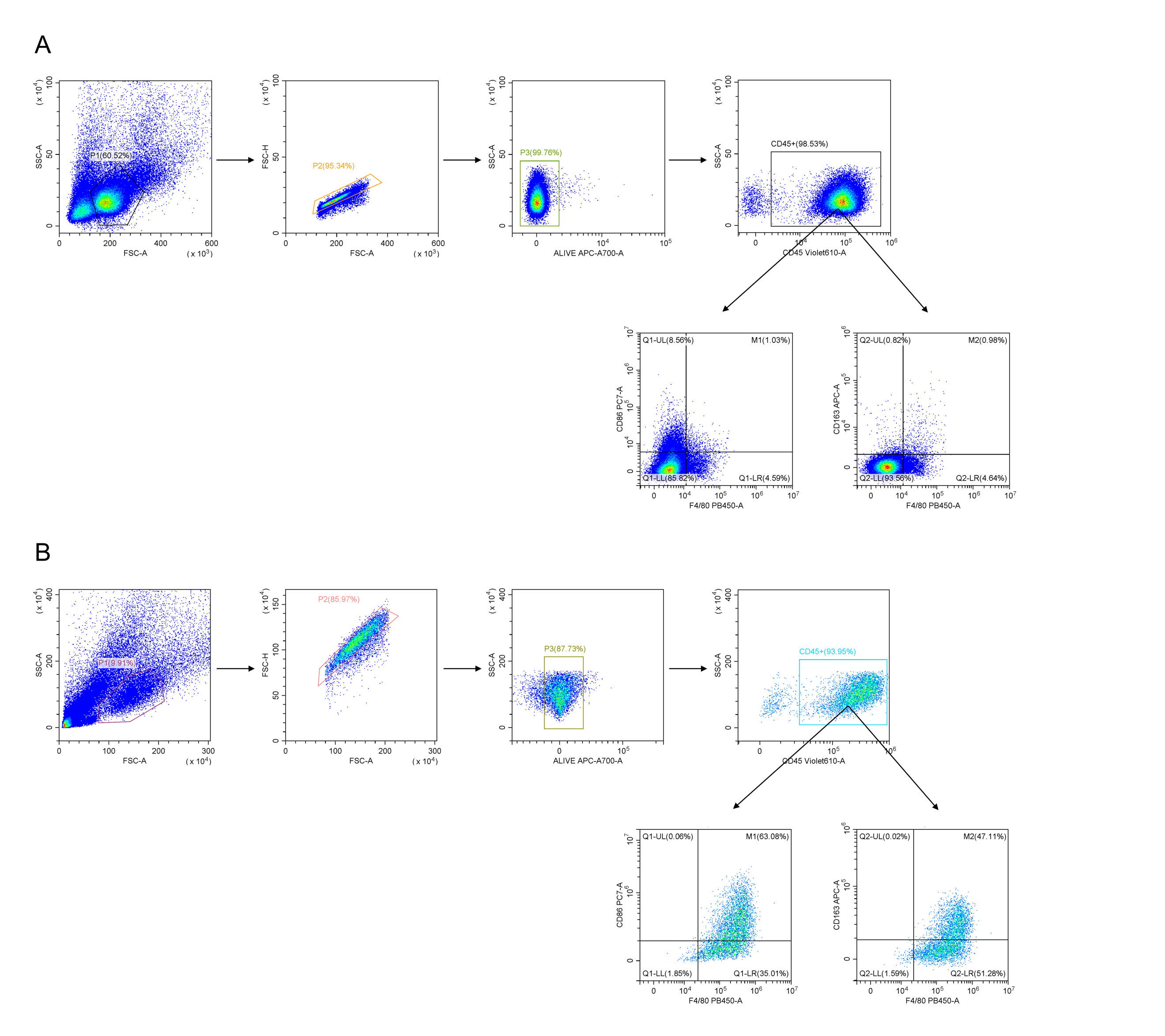
**

**Figure S9.** (A) The gating strategies of spleen/intestine macrophage cells. (B) The gating strategies of BMDMs.

**
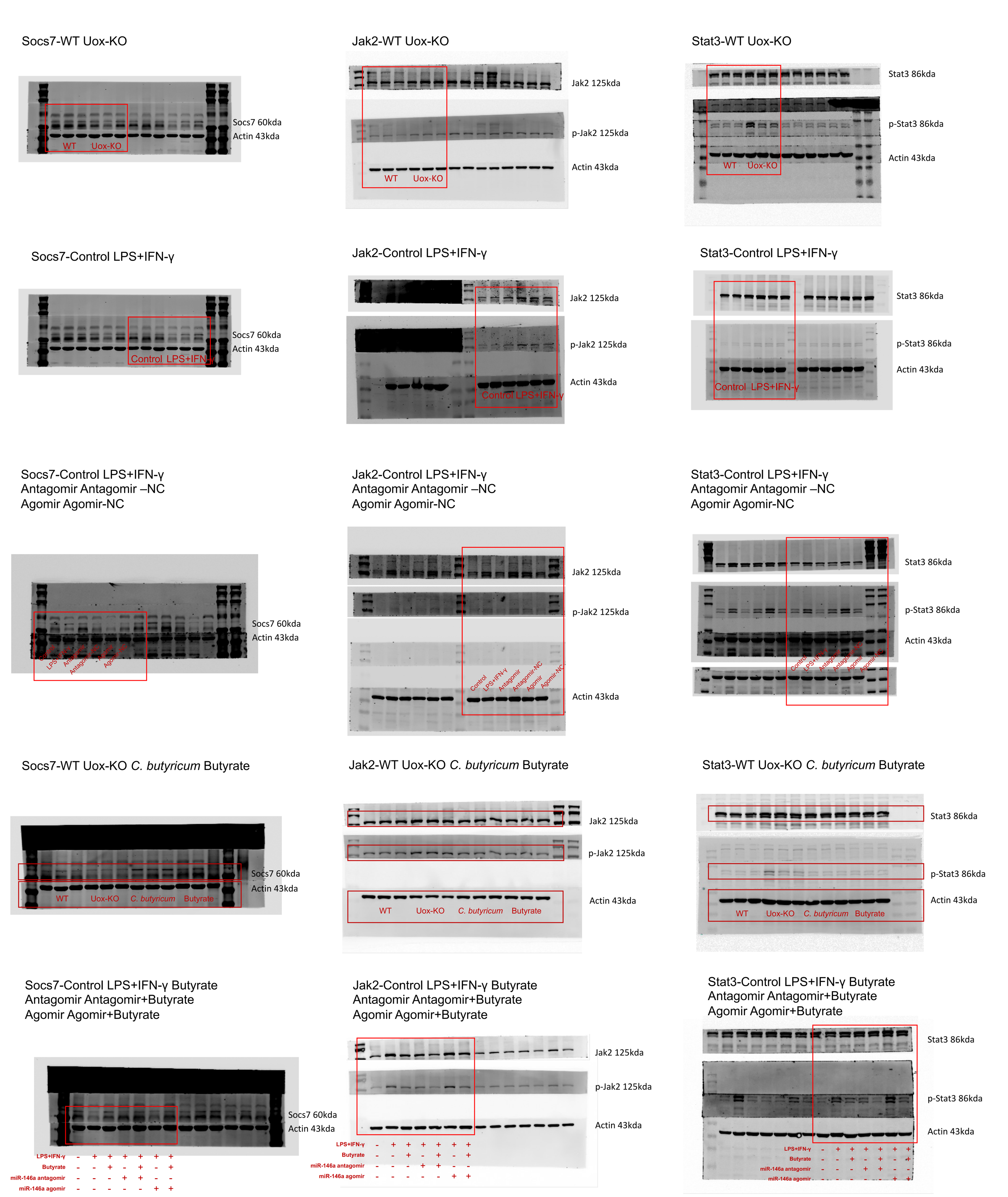

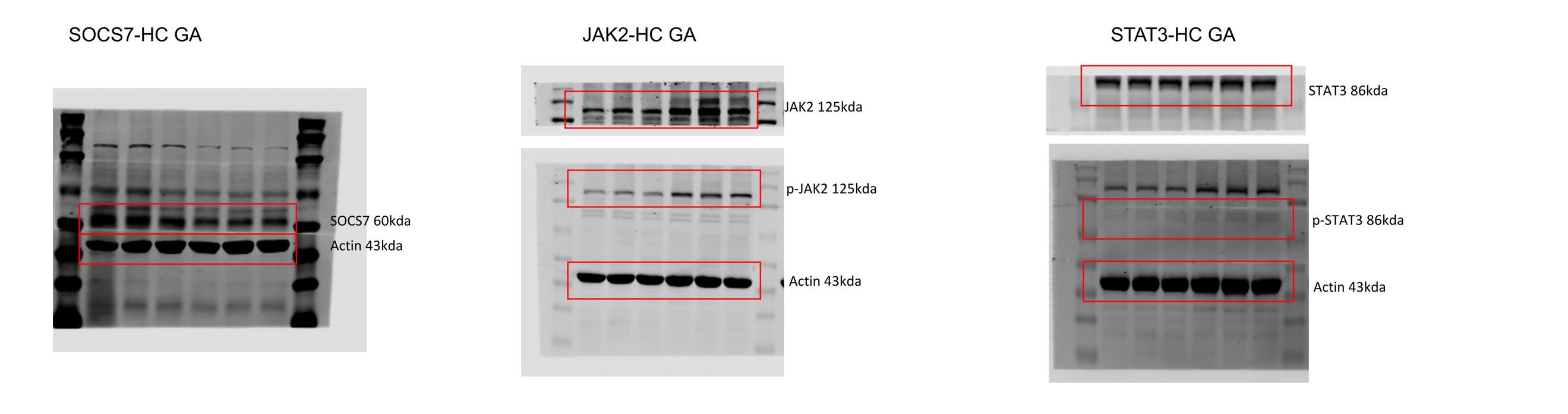
**

**Figure S10.** Original full length western blot.
